# Do alternative tobacco products induce less adverse respiratory risk than cigarettes?

**DOI:** 10.1186/s12931-023-02568-2

**Published:** 2023-10-31

**Authors:** Tariq A. Bhat, Suresh G. Kalathil, Noel Leigh, Alan Hutson, Maciej L. Goniewicz, Yasmin M. Thanavala

**Affiliations:** 1 Department of Immunology, Roswell Park Comprehensive Cancer Center, 665 Elm Street, Buffalo, NY 14263 USA; 2 Department of Health Behavior, Roswell Park Comprehensive Cancer Center, Buffalo, NY USA; 3 Department of Biostatistics and Bioinformatics, Roswell Park Comprehensive Cancer Center, Buffalo, NY USA; 4 Present Address: Department of Urology, Roswell Park Comprehensive Cancer Center, Buffalo, NY USA

**Keywords:** Heat-not-burn, IQOS; heated tobacco products, Electronic cigarettes; e-cigarette, Combustible cigarettes; smoking, Vaping, Immunity, Lung damage

## Abstract

**Rationale:**

Due to the relatively short existence of alternative tobacco products, gaps exist in our current understanding of their long-term respiratory health effects. We therefore undertook the first-ever side-by-side comparison of the impact of chronic inhalation of aerosols emitted from electronic cigarettes (EC) and heated tobacco products (HTP), and combustible cigarettes (CC) smoke.

**Objectives:**

To evaluate the potential differential effects of alternative tobacco products on lung inflammatory responses and efficacy of vaccination in comparison to CC.

**Methods:**

Mice were exposed to emissions from EC, HTP, CC, or air for 8 weeks. BAL and lung tissue were analyzed for markers of inflammation, lung damage, and oxidative stress. Another group was exposed for 12 weeks and vaccinated and challenged with a bacterial respiratory infection. Antibody titers in BAL and sera and pulmonary bacterial clearance were assessed.

**Main results:**

EC- and HTP-aerosols significantly augmented lung immune cell infiltrates equivalent to that achieved following CC-exposure. HTP and CC significantly increased neutrophil numbers compared to EC. All products augmented numbers of B cells, T cells, and pro-inflammatory IL17A^+^ T cells in the lungs. Decreased lung antioxidant activity and lung epithelial and endothelial damage was induced by all products. EC and HTP differentially augmented inflammatory cytokines/chemokines in the BAL. Generation of immunity following vaccination was impaired by EC and HTP but to a lesser extent than CC, with a CC > HTP > EC hierarchy of suppression of pulmonary bacterial clearance.

**Conclusions:**

HTP and EC-aerosols induced a proinflammatory pulmonary microenvironment, lung damage, and suppressed efficacy of vaccination.

**Supplementary Information:**

The online version contains supplementary material available at 10.1186/s12931-023-02568-2.

## Background

Tobacco use, particularly smoking of combustible cigarettes (CCs), remains a global public health problem and a key risk factor for disorders including respiratory diseases [1–10]. The tobacco industry innovatively commercialized alternative products like electronic cigarettes (e-cigarettes, ECs) and Heated Tobacco Products (HTPs) to reduce the harmful effects of inhaling toxic byproducts of tobacco combustion. These distinct product classes share common characteristics: (a) they are designed for inhalation, (b) they utilize electronic devices to produce nicotine-containing aerosols, and (c) they do not rely on combustion. A critical difference between those two types of alternative tobacco products is the source of nicotine: ECs aerosolize a solution containing nicotine but no tobacco leaf, whereas HTPs heat sticks containing actual tobacco (Table 1).

**Table 1:** Comparison of product performance characteristics and primary ingredients of combustible cigarettes (CC), Heated Tobacco Products (HTP), and e-cigarettes (EC)

	Combustible cigarettes (CC)	Heated tobacco products (HTP)	E-cigarettes (EC)
Nicotine	Yes	Yes	Yes (in most products*)
Tobacco	Yes	Yes	No (nicotine in a form of liquid solution)
Combustion	Yes	No (a potential risk of incomplete combustion)	No (a potential risk of thermal degradation of nicotine solution ingredients)
Temperature	Yes (very high during puffs)	Yes (generally lower than in combustible cigarettes)	Yes (generally lower than in combustible cigarettes)
Electronic system	No	Yes	Yes
Example of the product			

*Some brands of EC are available in a nicotine-free version

Manufacturers of both types of alternative tobacco products center their marketing strategies around purportedly lower health risks than CC. Those reduced health risk claims are primarily based on reductions in toxicant levels in emissions from ECs and HTPs compared to CCs. For example, industry-funded studies have shown the absence of numerous combustion by-products (including CO and 1,3-butadiene) in aerosols emitted by HTPs [11–14]. However, because HTP still contains tobacco, several tobacco-related toxicants (e.g. cancer causing tobacco-specific nitrosamines, TSNAs) have been found in emissions from those products. Numerous independently funded studies have shown that aerosols emitted from ECs also do not contain combustion by-products and additionally do not contain tobacco-related toxicants [11–14]. However, chronic use of EC or HTP still results in repeated inhalation of respiratory toxicants. HTPs emit levels of respiratory toxicants intermediate between those found in emissions form ECs and CCs [15–18]. Deliver concerns have been raised about the potential unique respiratory health risks of EC use, including the effects of inhaled flavorings, nicotine solvents, and their thermal breakdown products [19–21].

The respiratory system responds to foreign agents, including inhaled smoke by initiating a process of inflammation [22–24]. Amid this protective response, respiratory epithelial cells are activated to induce damage-associated molecular patterns and proinflammatory cytokines and chemokines, which serve as chemoattractants for various immune cells [23]. The overall milieu generated is very immunosuppressive and results in a diminution of immune response to vaccination and pulmonary infection [7–10]. This complex response has been well characterized for tobacco smoke, however little is known if similar responses are elicited by alternative tobacco products (e.g. infiltration and activation of the same immune cell subsets to the lung, release of similar cytokines). If the responses elicited are indeed similar, then the question remains if the magnitude of the changes is also equivalent to tobacco smoke. If the responses seen by exposures to EC and HTPs are weaker compared to tobacco smoke, this could suggest a potential reduction in health risk in smoker switching to ATPs.

Due to the relatively short existence of alternative tobacco products, data on their long-term respiratory health effects are currently unavailable. In the interim, evidence from animal studies may provide crucial information on the potential adverse risks of these emerging tobacco products. Recognizing the knowledge gap in the field, we undertook the first-ever side-by-side comparison of the impact of chronic inhalation of aerosols emitted from EC and HTP, and CC smoke to determine if a hierarchy exists in their potential to induce detrimental pulmonary effects and to suppress immunity.

## Materials and methods

### Overview of the study protocol

**Fig. 1:**
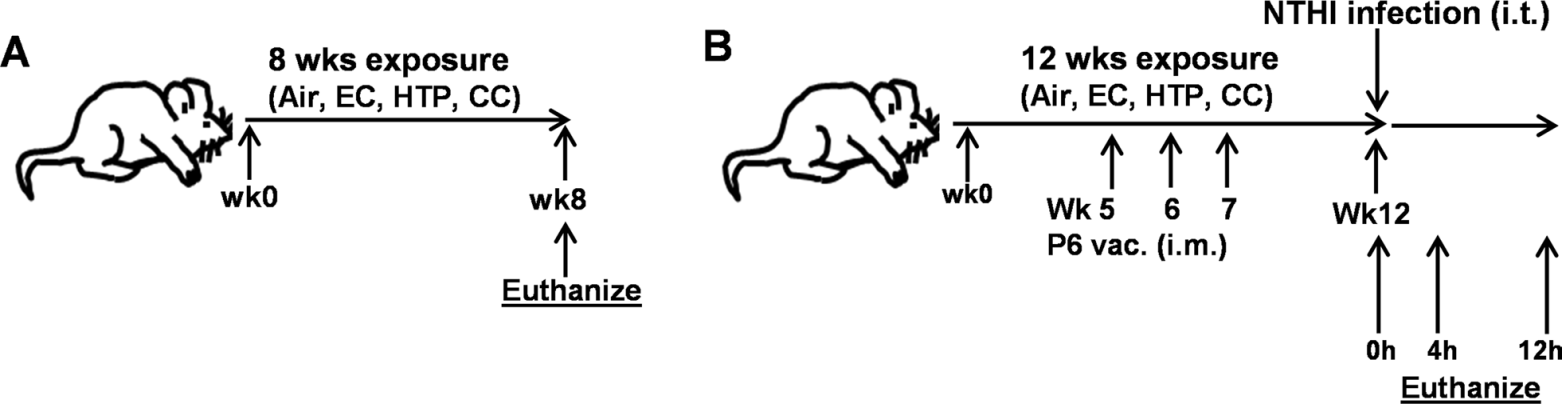
Schema of 8-week and 12-week animal exposures. Mice were exposed to aerosols from 3 products for 8 weeks (**A**) or 12 weeks (**B**). Air-exposed animals served as control for each group. Mice exposed for 12 weeks were vaccinated i.m. at wk5, wk6 and wk7 after the start of exposures, and were given acute intratracheal challenge with NTHI (10^6^ cfus/mouse in 50uL PBS) at the end of the wk12 for 0, 4 and 12 h

Using an animal exposure model, we compared the impact of chronic inhalation of EC, HTP, and CC emissions on lung inflammation and immunity. Mice were exposed to emissions from EC, HTP, CC, or air (control) (Fig. 1). At week 8 after exposures, one group of animals (Fig. 1A; n = 10 for air, n = 20 for EC, HTP, and CC/group) were sacrificed, and BAL and lung tissue were collected and analyzed for markers of immune response in lungs, lung damage, and oxidative stress. A second group of animals (Fig. 1B; n = 20 for air, EC, HTP, and CC/group) received intramuscular (i.m.) prophylactic vaccination against a respiratory pathogen at weeks 5, 6, and 7. Vaccination efficacy was measured by quantifying antigen-specific antibody titers in serum (weeks 5–12) and in BAL at euthanasia. Finally, all vaccinated animals were challenged at week 12 with a respiratory pathogen and bacterial clearance from the lungs and lung damage were assessed immediately after intratracheal challenge, 4 and 12 h later. Details of tobacco products, assessment of pulmonary inflammation, lung damage, markers of oxidative stress, quantification of myeloperoxidase activity (MPO) and neutrophil elastase (NE), and vaccination efficacy are provided in Additional file 1.

### Animal exposure conditions

Animal exposure conditions are provided in detail in Additional file 1. We decided to expose animals to an equivalent dose of nicotine delivered from all tested products. Nicotine equivalency was determined by quantifying serum cotinine levels (a nicotine metabolite) in blood samples collected 30 min post-exposure. Despite differences in the puffing protocols used in our experiments, we achieved equivalent exposure to nicotine from all tested products (Table 2).

**Table 2:** Exposure conditions to emissions from EC, HTP, and CC and air (control)

	Air	EC	HTP	CC
Puffing protocol				
Number of products used over 5 h	–	Approx. 0.5 ml	20 tobacco sticks	30 cigarettes
Number of puff clusters over 5 h	13	13	20	30
Number of puffs per cluster	11	11	12	8
Total number of puffs taken over 5 h	143	143	240	240
Interval between puff clusters	20 min	20 min	9 min	6 min
Airborne exposure				
Airborne nicotine (µg/m^3^)	17.7 ± 13.6	336.7 ± 86.3	649.5 ± 262.2	1097.0 ± 361.7
PM_5.0_ (mg/m^3^)	< LOQ	39.0 ± 18.6	18.6 ± 5.7	34.8 ± 16.0
TPM (mg/m^3^)	< LOQ	302.0 ± 127.4	18.1 ± 7.5	292.4 ± 72.5
Thirdhand exposure				
Nicotine deposited on surface (mg/m^2^/5 h)	< LOQ	< LOQ	< LOQ	< LOQ
Nicotine deposited on fur (mg/m^2^/8 wks)	< LOQ	< LOQ	< LOQ	< LOQ
Biomarker of nicotine exposure				
Serum cotinine in males (ng/ml)	< LOQ	32.2 ± 10.0*	34.8 ± 10.3*	33.5 ± 10.0*
Serum cotinine in females (ng/ml)	< LOQ	52.7 ± 30.1*	53.6 ± 6.6*	56.3 ± 28.5*

< LOQ = below the limit of quantitation (LOQs were as follows: airborne nicotine 0.4 µg/m^3^; PM_5.0_ 4.0 mg/m^3^; TPM 0.001 mg/m^3^; surface nicotine 1.25 mg/m^2^; nicotine deposited on fur 31.3 mg/m^2^; cotinine 2.0 ng/ml). * No significant changes were found between exposure conditions (*p* < 0.05; Kruskal–Wallis non-parametric test with Dunn’s multiple comparisons)

### Statistical analysis

Statistical analyses performed were similar to those presented in our recently published paper [25]. Due to the relatively small samples size, statistically significant differences between the mean rank values of different exposure groups (EC, HTP, CC and air controls) were determined by performing Kruskal–Wallis’s non-parametric test. *P* values were corrected for multiple testing using the ‘two-stage linear step-up procedure of Benjamini, Krieger and Yekutieli’ false discovery rate (FDR) method and the differences between two groups were considered statistically significant at p < 0.05 when FDR was set at Q < 0.1. We also evaluated if there were differences between male versus female mice in the responses to inhalation of CBD and nicotine aerosols in comparison with air. All statistical analyses were carried out using GraphPad Prism 9.5.1 software (GraphPad; La Jolla, California, USA). Data are shown as bar diagrams with mean ± SE. For Figs. 7 and 8, to assess differences between the mean values of different exposure groups (EC, HTP, CC, and air), we performed a 2-way ANOVA with Tukey's post-test comparisons using GraphPad Prism 9.5.1 software.

We excluded 5 animals that died during the study for reasons unrelated to the experimental exposures, considering them missing at random and did not include them in the statistical analysis. We evaluated differences between male and female mice using a similar approach.

## Results

### Chronic inhalation exposure to aerosols emitted from alternative tobacco products resulted in the accumulation of innate and adaptive immune cells in the lungs

**Fig. 2:**
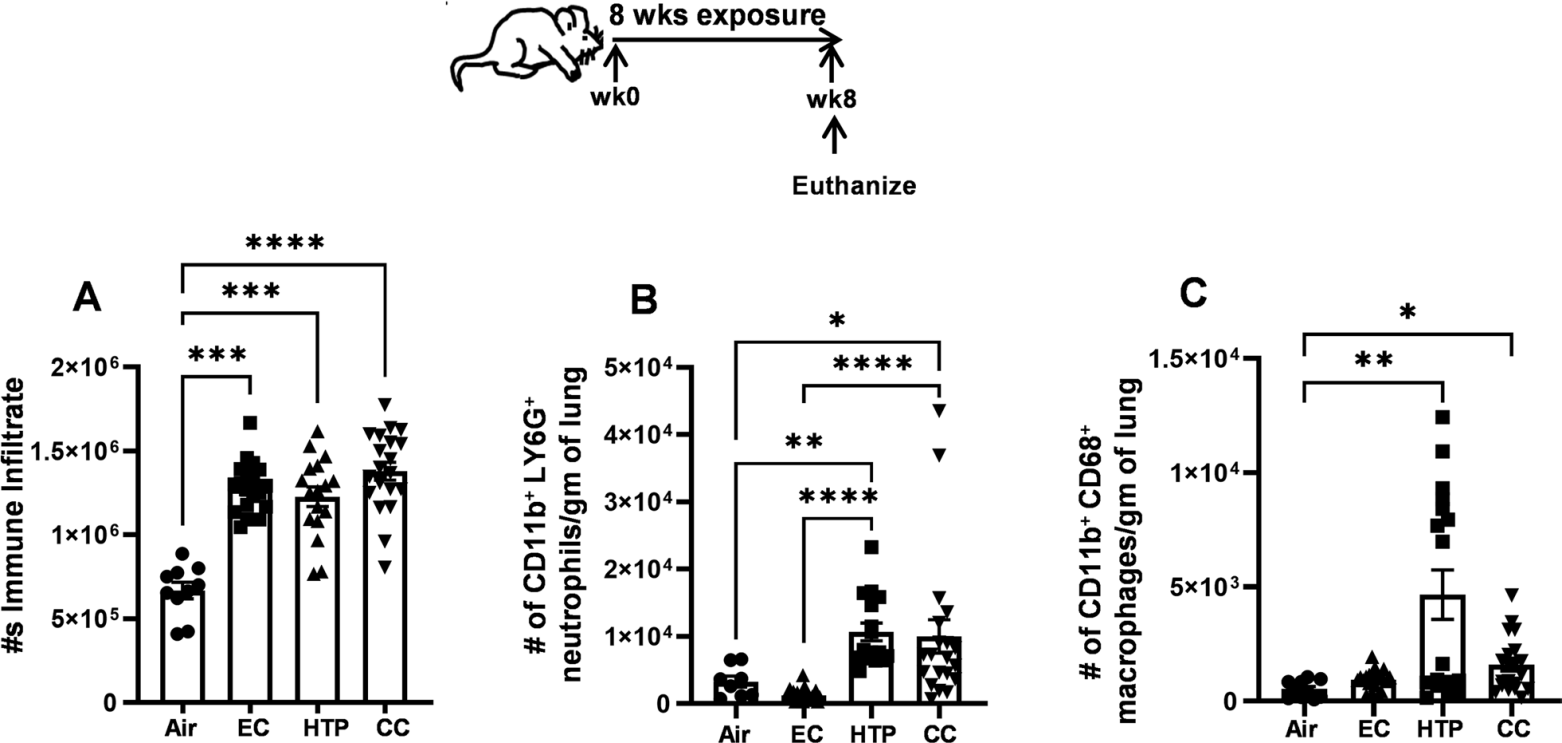
Chronic inhalatory exposure to aerosols emitted from alternative tobacco products modulates lung innate immune-cell accumulation. Total numbers of leukocytes (**A**), CD11b^+^Ly6G^+^ neutrophils (**B**), CD11b^+^ CD68^+^ macrophages in the lungs of mice exposed to air, EC, HTP or CC were determined by multicolor flow cytometry using specific antibody markers. Staining protocol and gating strategy are described previously [47] and depicted in Additional file 1: Fig. S1 and with details in online Additional file 1. Data are shown as bar diagrams with mean ± SE. Non-parametric Kruskal–Wallis test with FDR correction for multiple comparison was performed to see if statistically significant differences exist between two groups using GraphPad Prism V.9 software (GraphPad; La Jolla, California, USA). Difference between two groups is considered significant at p < 0.05 and are indicated with symbols **p < 0.01; ***p < 0.001; ****p < 0.0001 using a post-test comparison with Tukey’s correction. In each exposure condition, n = 10 for air (5M + 5F) and n = 20 for EC, HTP, and CC (10M + 10F) per group were used

Chronic inhalation exposure to EC and HTP induced a significant augmentation of total immune cell infiltrates in the lungs compared to air (Fig. 2A), equivalent to that achieved following CC exposure. Following HTP exposure, male mice showed significantly more immune-cell infiltration than female mice (Additional file 1: Fig. S2A). HTP- and CC-induced augmentation in the number of neutrophils in the lungs was significantly more compared to EC (*p* < 0.0001) (Fig. 2B). The augmentation of neutrophils following HTP exposure was similar in magnitude to that found after CC exposure, with no sex-related differences (Additional file 1: Fig. S2B).

HTP and CC but not EC significantly augmented the numbers of CD11b^+^ CD68^+^ macrophages in the lung, compared to air (*p* < 0.05) (Fig. 2C). We observed sex-related differences in the augmentation of CD11b^+^CD68^+^ macrophages in the lungs of male mice following HTP and CC exposures (Additional file 1: Fig. S2C).

**Fig. 3:**
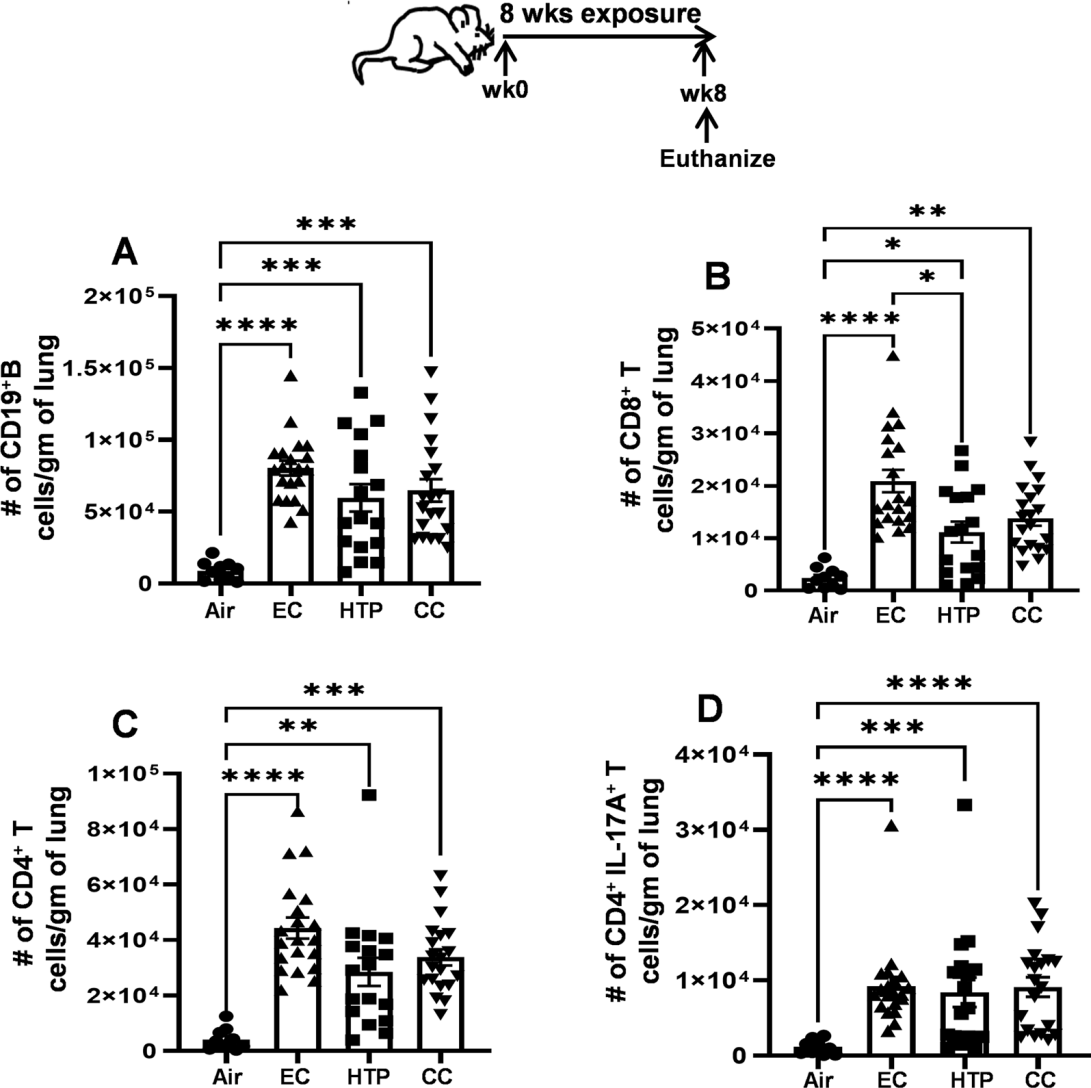
Chronic exposure to alternative tobacco product-aerosols induces adaptive and proinflammatory immune-cell accumulation in the lungs. Total numbers of CD19^+^ B cells (**A**), CD8^+^ T cells (**B**), CD4^+^ T cells (**C**) and CD4^+^IL17A^+^ inflammatory T cells in the lungs of mice exposed to air, EC, HTP or CC were calculated using multicolor flow cytometry after staining with specific antibody markers. Gating strategy was similar to that described previously [47] and depicted in Additional file 1: Fig. S1. Data are given as bar diagrams with mean ± SE. Non-parametric Kruskal–Wallis test with FDR correction for multiple comparison was performed to see if statistically significant differences exist between two groups using GraphPad Prism V.9 software (GraphPad; La Jolla, California, USA). Difference between two groups is considered significant at p < 0.05 and are indicated with symbols *p < 0.05, **p < 0.01; ***p < 0.001; ****p < 0.0001 using a post-test comparison with Tukey’s correction. In each exposure condition, we used n = 10 animals for air (5M + 5F) and n = 20 animals for EC, HTP, and CC (10M + 10F) per group

The numbers of CD19^+^ B cells recruited to the lung were equivalent following EC, HTP, and CC exposures (Fig. 3A) and markedly augmented compared to air (*p* < 0.001). All three products significantly increased the numbers of CD8^+^ T cells in the lungs compared to air; however, this was greater after EC exposure compared to HTP (*p* < 0.05) (Fig. 3B). CD8^+^ T cell numbers following HTP and CC exposures were equivalent. Aerosol inhalation from EC and HTP products induced significant augmentation of CD4^+^ T cell numbers in the lungs compared to air(*p* < 0.01), and this augmentation was similar in magnitude as observed following CC exposure (Fig. 3C). The numbers of CD4^+^ T cells following EC and HTP exposures were not significantly different from those observed following CC exposure. While increased numbers of B cells were detected in the lungs of male compared to female mice following HTP and CC exposures, CD8^+^, and CD4^+^ T cells were augmented in male mice only after HTP-exposure (Additional file 1: Fig. S3A–C). Infiltration of pro-inflammatory CD4^+^IL17A^+^ T cells to the lungs was significantly greater following exposure of all three products compared to air (*p* < 0.001) (Fig. 3D). Male mice had significantly increased numbers of CD4^+^IL17A^+^ T cells after HTP and CC exposures (Additional file 1: Fig. S3D).

### Chronic exposure to aerosols emitted from alternative tobacco products modulated proinflammatory cytokines and chemokines levels

**Fig. 4:**
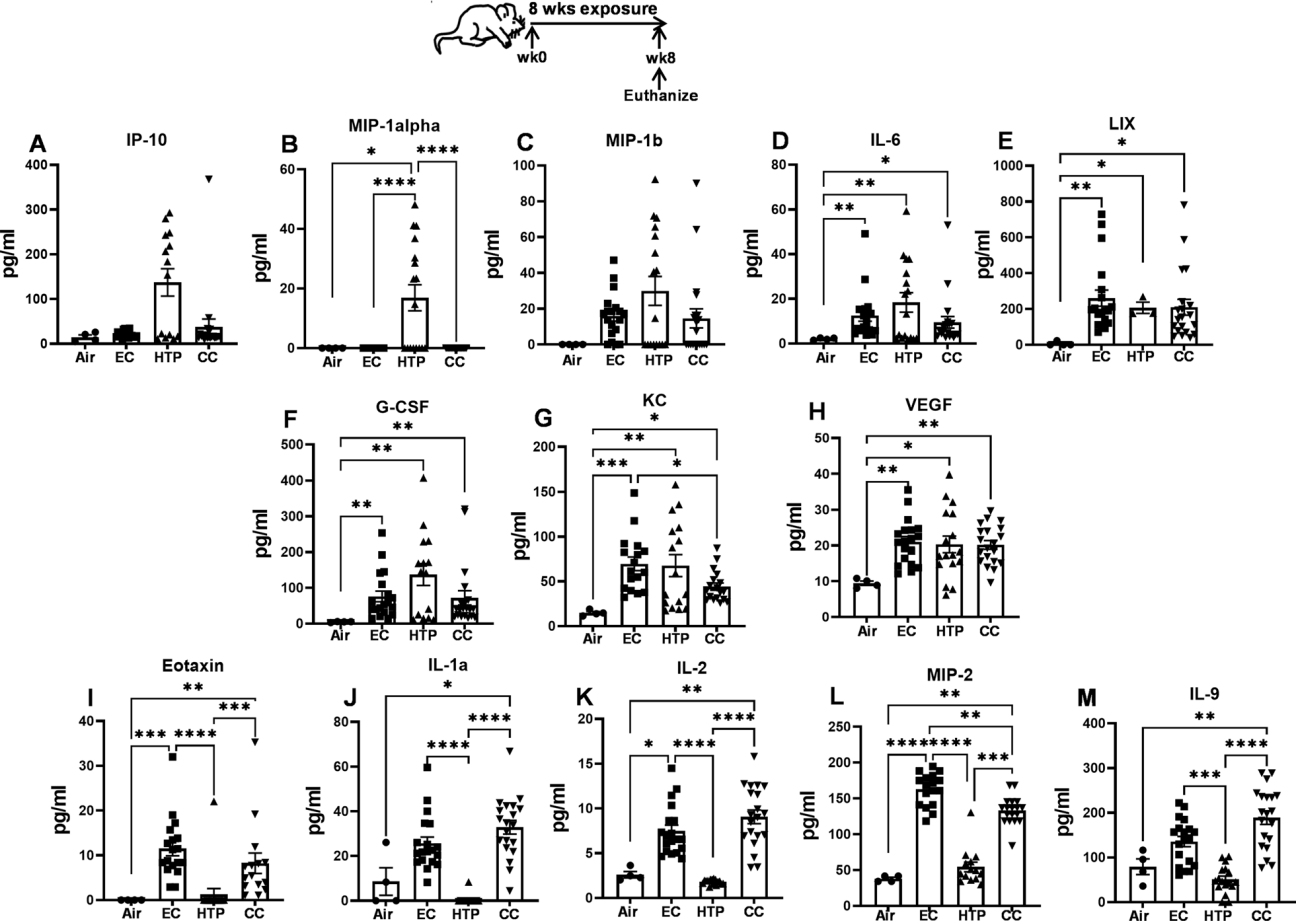
Chronic exposure to alternative tobacco product aerosols modulated pulmonary proinflammatory cytokine and chemokine levels. Levels of inflammatory cytokines and chemokines in the BAL (**A**–**M**) of mice were measured at end of the 8-week exposure to air, EC, HTP or CC aerosols using MILLIPLEX MAP Kit as described previously [47] and in detail in Additional file 1: supplemental methods. Data are presented as bar diagrams with mean ± SE. Non-parametric Kruskal–Wallis test with FDR correction for multiple comparison was performed to see if significant differences exist between groups using GraphPad Prism V.9 (GraphPad; La Jolla, California, USA). Difference between two groups is considered significant at p < 0.05 and indicated with symbols *p < 0.05; **p < 0.01; ***p < 0.001; ****p < 0.0001. For each exposure condition, n = 10 animals for air (5M + 5F) and n = 20 animals for EC, HTP, and CC (10M + 10F) per group

Overall, the levels of various inflammation-associated cytokines and chemokines in the BAL were modulated after exposure to each product. While increased IP10, and MIP-1β were detected in the BAL after HTP exposure compared to air, the differences were not statistically significant (Fig. 4A, C). Chronic exposure to HTP, but not to EC, elevated MIP-1α when compared to air (*p* < 0.05), and their levels after HTP exposure were markedly higher than following EC (*p* < 0.0001) and CC exposures (*p* < 0.0001) (Fig. 4B). IL6, LIX, G-CSF, KC and VEGF levels were augmented following exposure to all three products compared to air (*p* < 0.05) (Fig. 4D–H) with no differences when comparing EC, HTP, and CC, except for KC, where these levels following EC exposure were higher compared with CC exposure (*p* < 0.05). Compared to air, augmented levels of eotaxin, IL-1α, IL-2, and MIP-2 were found in BAL following exposure to EC but not HTP. Levels measured in the BAL of the EC group were significantly higher compared to HTP (*p* < 0.0001) (Fig. 4I–L). The levels of eotaxin, IL-1α, IL-2 and MIP-2 were significantly lower following HTP than CC exposure (*p* < 0.001). The levels of IL-9 following EC and HTP exposures were unchanged when compared to air but were significantly lower following HTP compared to EC and CC exposures (*p* < 0.001) (Fig. 4M).

### Inhalation of HTP and EC aerosols resulted in lung damage

**Fig. 5:**
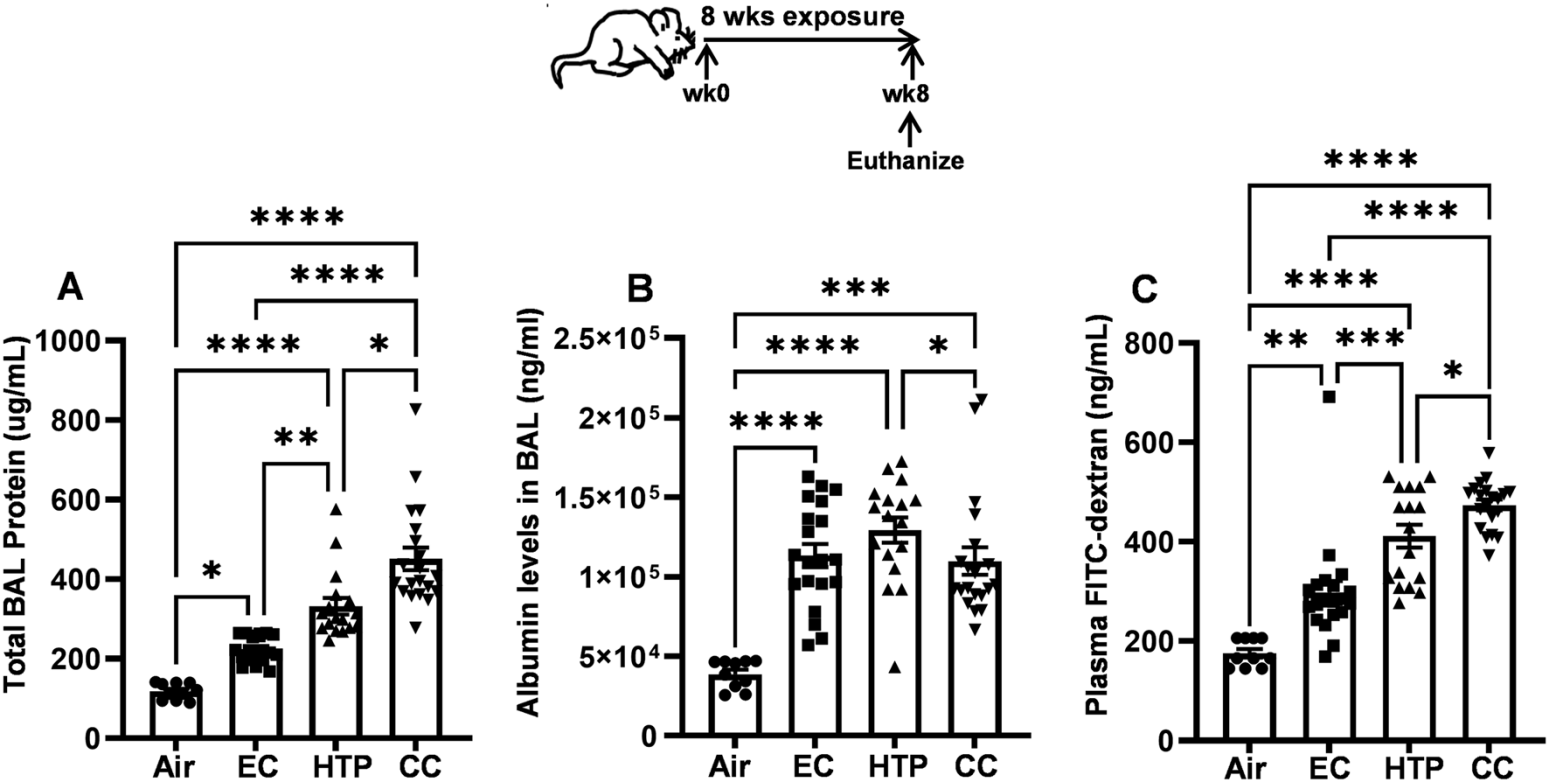
Chronic inhalatory exposure to aerosols from alternative tobacco products induces markers of lung damage. At the end of the 8-week exposures, mice were euthanized, BAL harvested and the levels of total proteins (**A**), albumin (**B**) in the BAL, and the levels of FITC-dextran (**C**) leaking into plasma were quantified as described previously [47] and given in detail in Additional file 1. Results are shown as bar diagrams with mean ± SE. Differences between groups is considered significant at p < 0.05 and are indicated as symbols *p < 0.05; **p < 0.01; ***p < 0.001; ****p < 0.0001, calculated after performing non-parametric Kruskal–Wallis test with FDR correction for multiple comparisons by employing GraphPad Prism V.9 software (GraphPad; La Jolla, California, USA). n = 10 animals for air (5M + 5F) and n = 20 animals for EC, HTP, and CC (10M + 10F)/group were used for each exposure condition

**Fig. 6:**
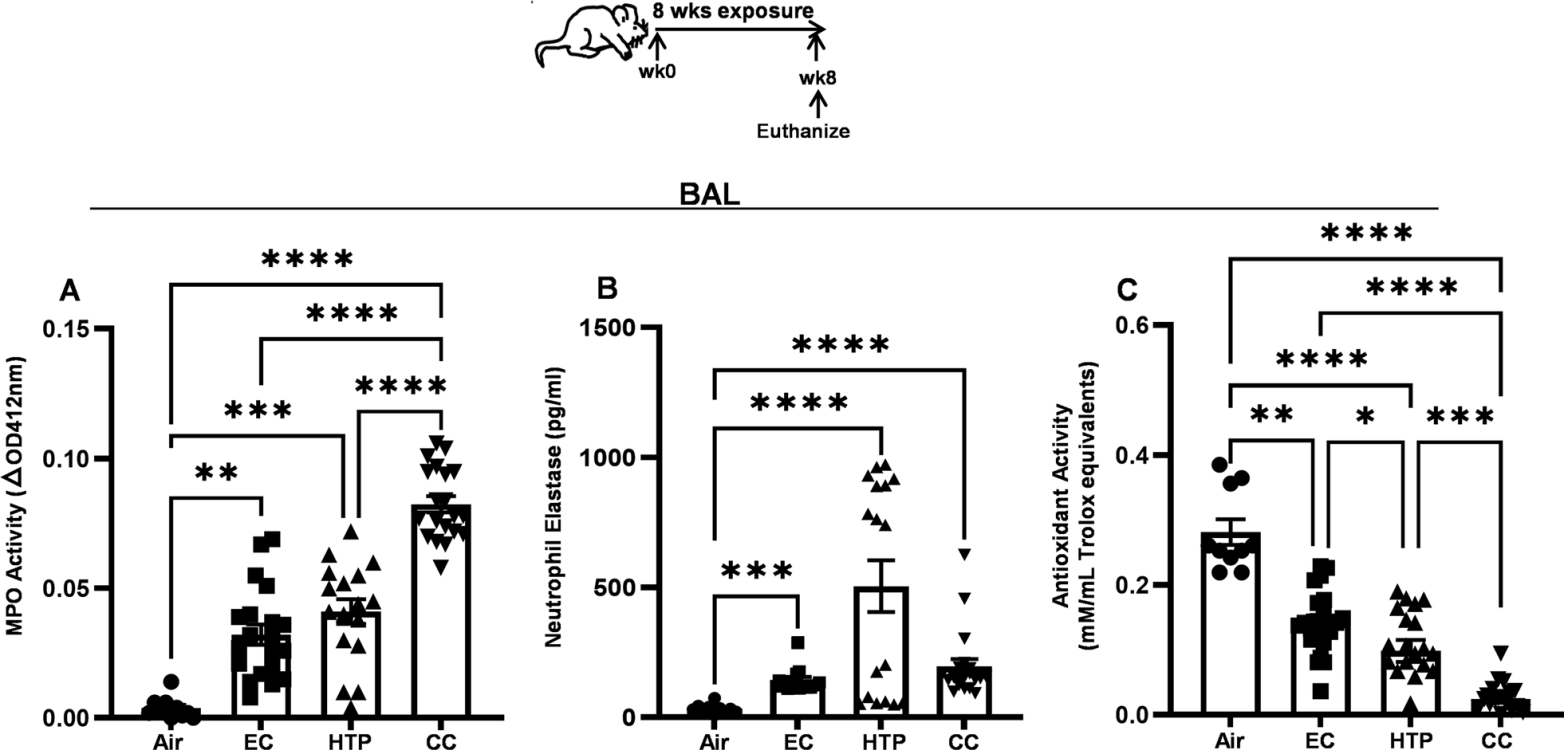
Chronic inhalatory exposures to EC, HTP and CC aerosols induce lung damage. Mice exposed to aerosols from alternative tobacco products and CC for 8 weeks were euthanized and BAL harvested. MPO activity, levels of NE and antioxidant potential in the BAL were measured as described previously [47] and details provided in Additional file 1. Data are presented as bar diagrams with mean ± SE. Difference between two groups is considered significant at p < 0.05 and is indicated by symbols *p < 0.05, **p < 0.01, ***p < 0.001 ****p < 0.0001, calculated after performing a non-parametric Kruskal–Wallis rank test with FDR correction for multiple comparisons by employing GraphPad Prism V.9 software (GraphPad; La Jolla, California, USA). We used n = 10 animals for air (5 M + 5F) and n = 20 animals for EC, HTP, and CC (10M + 10F) per group for each exposure condition

The levels of total proteins in the BAL were significantly increased following CC, HTP, and EC exposures compared to air, with CC > HTP > EC > air hierarchy (Fig. 5A). All products equivalently augmented the leak of albumin into the bronchoalveolar space of mice compared to air (*p* < 0.001), and this leak following HTP exposure was significantly more than after CC exposure (*p* < 0.05) (Fig. 5B). The levels of FITC-dextran in the plasma of mice exposed to EC, HTP, and CC were significantly higher than air (*p* < 0.01) (Fig. 5C). Additionally, while the plasma FITC-dextran levels were significantly greater following HTP and CC vs. EC exposure (*p* < 0.001), these levels were higher when comparing CC vs HTP groups (*p* < 0.05) (Fig. 5C). Only sex-based difference observed in these lung damage marker levels was higher levels of albumin in the BAL of female mice following EC exposure (Additional file 1: Fig. S4A–C). Chronic exposure to HTP and EC augmented MPO activity in the BAL compared to air (*p* < 0.01) (Fig. 6A). When compared to CC, the MPO activity induced following HTP (*p* < 0.001) or EC exposure (*p* < 0.0001) was lower (Fig. 6A), and no sex-based differences were noted (Additional file 1: Fig. S5A). NE levels in the BAL were markedly higher following exposure to all three products when compared to air exposure (*p* < 0.001) (Fig. 6B). Significantly elevated NE levels were noted in male compared with female mice following exposure to HTP (Additional file 1: Fig. S5B).

### Exposure to aerosols emitted from alternative tobacco prducts decreased lung antioxidant activity

The decreased antioxidant activity resulting from exposure to all products was significant compared to air (*p* < 0.01) (Fig. 6C); however, the suppression of antioxidant activity was maximal after exposure to CC compared to EC and HTP (*p* < 0.001) (Fig. 6C). Additionally, antioxidant activity following HTP exposure was significantly more suppressed than following EC exposure (*p* < 0.05). No sex-based differences were observed in antioxidant activity following any of these exposures (Additional file 1: Fig. S5C).

### Chronic exposure to alternative tobacco product aerosols diminished the efficacy of vaccination

**Fig. 7:**
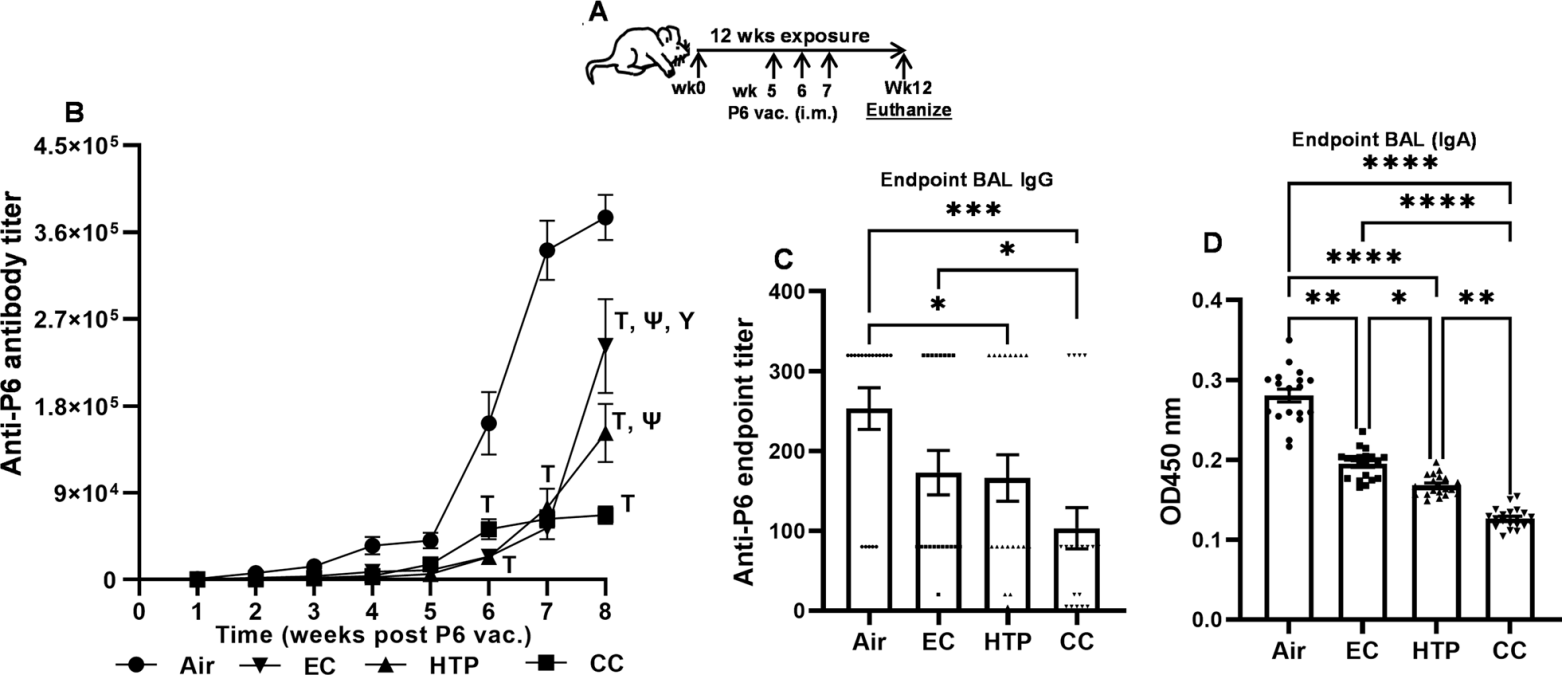
Chronic exposure to alternative tobacco product aerosols suppresses development of antibody responses to vaccination. Animals exposed to air, EC, HTP, and CC received prophylactic P6 Ag vaccination i.m. against a respiratory pathogen at weeks 5, 6, and 7 after exposures were started as shown in schema (**A**). Vaccination efficacy was measured by quantifying antigen-specific antibody titers in serum (weeks 5–12) and in BAL at euthanasia. Weekly serum was collected from aerosol-exposed, P6 immunized mice as described in Additional file 1 and total anti-P6 Ig levels were measured in weekly serum (**B**) and endpoint BAL (**C**) samples. To quantify mucosal IgA Ab levels (**C**) in the BAL of mice, the OD values at 450 nm were measured using BAL dilutions at 1:400 in P6-specific ELISA as described previously [10] and provided in detail in Additional file 1: supplemental methods. Data are shown as curve (**B**) or as bar diagram with individual data sets (**C**, **D**) and given as mean ± SE. Two-way ANOVA with Tukey’s post-test multiple comparisons (**A**) or non-parametric Kruskal–Wallis test with FDR correction for multiple comparison (**C**, **D**) was performed to determine statistically significant differences between two groups by GraphPad Prism 9.5.1 software (GraphPad; La Jolla, California, USA). Difference between two groups was considered significant at p < 0.05, and symbols ***p < 0.001; ****p < 0.0001 are used to denote significant difference between two groups. For, two-way ANOVA, symbols denote as follows: ^┬^p < 0.0001 vs Air; ^Ψ^p < 0.0001 vs CC; ^Υ^p < 0.001 vs IQOS. n = 20 for air, EC, HTP, and CC (10 M + 10F) per group for each exposure condition

When aerosol-exposed mice were vaccinated with P6 Ag (Fig. 7A), we noted that the kinetics of the appearance of serum anti-P6 antibodies following exposures to all products were slower, and the magnitude of antibody titers accumulating in the sera of mice from wk4 onwards was substantially lower compared with air-exposed mice (Fig. 7B, *p* < 0.0001). While the titers of anti-P6 antibodies in the serum of vaccinated mice exposed to HTP or EC were equivalent from weeks 1 to 7, the end-point antibody titers at wk8 post-vaccination were significantly more suppressed in the serum of HTP-exposed compared with EC-exposed mice (p < 0.001) (Fig. 7B). Antibody titers in CC-exposed mice were suppressed at week 8 compared to both alternative tobacco products (*p* < 0.0001) (Fig. 7B).

End-point anti-P6 titers after vaccination were significantly lower in the BAL of mice exposed to only HTP and CC compared to air-exposed control (*p* < 0.05) (Fig. 7C). Additionally, all products significantly decreased P6-specific mucosal IgA antibody levels in the BAL of P6-vaccinated mice compared to air (*p* < 0.01) (Fig. 7D). The levels of anti-P6 IgA antibodies were lower following HTP vs*.* EC exposure (*p* < 0.05) but maximally suppressed following CC compared to both EC and HTP exposures (*p* < 0.01) (Fig. 7D).

**Fig. 8:**
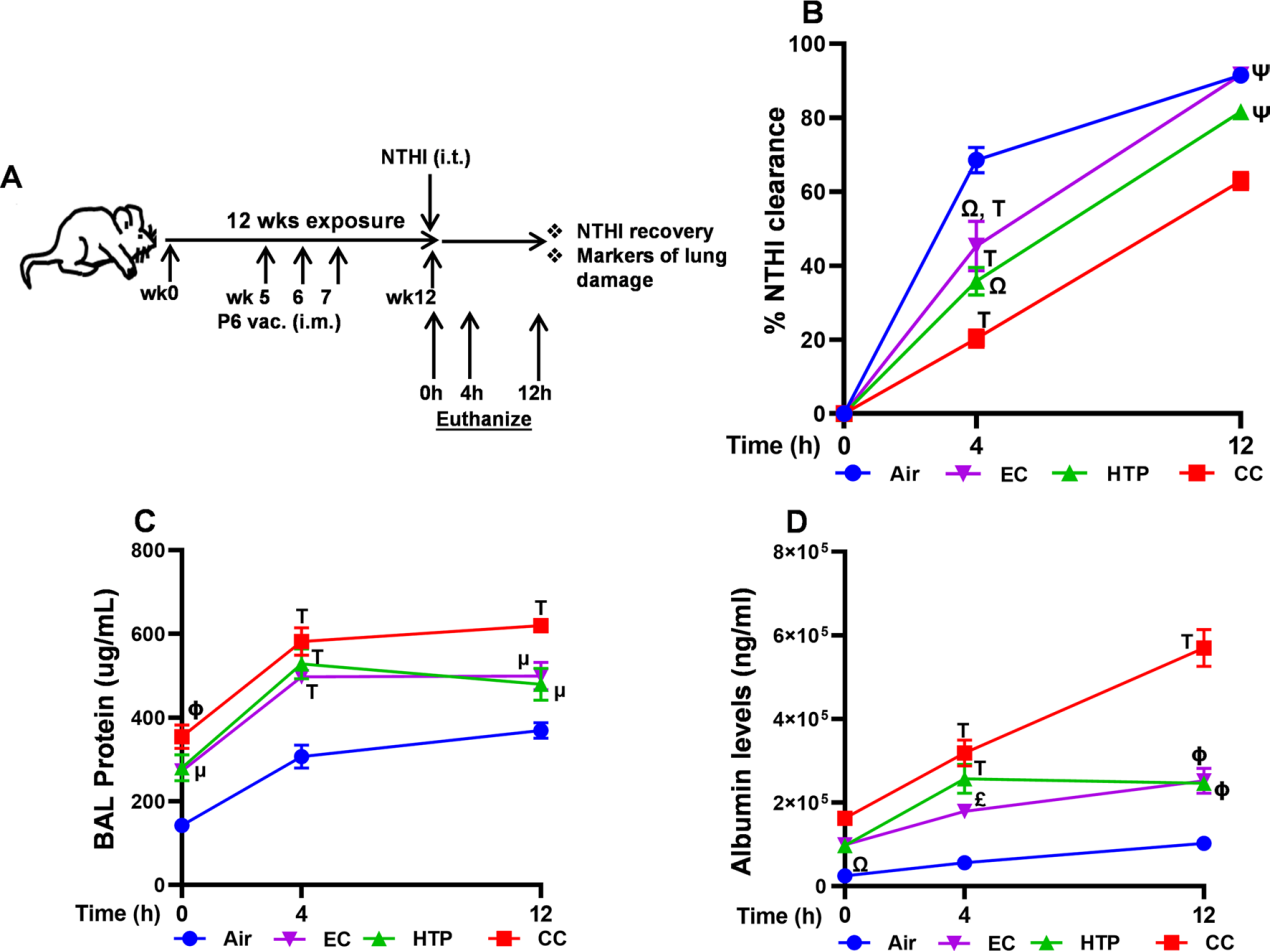
Chronic alternative tobacco product aerosol-suppressed vaccination efficacy translates to diminished bacterial clearance from the lungs. All exposed-mice were given an intratracheal instillation of 1 million NTHI cfu 8 weeks after P6 vaccination. Animals were euthanized 0, 4 and 12 h after bacterial challenge and BAL and lungs were harvested. Lung tissue homogenates were prepared to measure %NTHI clearance, and the BAL was processed to quantify the markers of lung damage as shown in schema (**A**) and described in detail in Additional file 1. Lung bacterial burden was calculated as NTHI clearance from the lungs of mice and measured by bacterial colony-plating assay. Data are represented as %NTHI clearance (**B**). Levels of total proteins (**C**) and albumin (**D**) in the BAL were quantified as described in Additional file 1: supplementary methods. Two-way ANOVA was performed to determine statistically significant differences between two groups and p-values were calculated using Tukey’s post-test multiple comparison by GraphPad Prism 9.5.1 software (GraphPad; La Jolla, California, USA). Difference between two groups was considered significant at p < 0.05, and symbols ^μ^p < 0.05 vs*.* Air; ^£^p < 0.01 vs*.* Air; ^Φ^p < 0.001 vs*.* Air; ^┬^p < 0.0001 vs*.* Air; ^Ω^p < 0.01 vs*.* CC; ^Ψ^p < 0.0001 vs*.* CC and ^Υ^p < 0.001 vs*.* IQOS are used to denote significant difference between two groups. Number of mice was n = 6 at 0 h, n = 8 at 4 h and n = 6 at 12 h per group; mean ± SEM

To test whether this reduction in vaccine-induced systemic and mucosal immunity translates to a delay and reduction in the ability of these mice to clear an acute respiratory infection, we intratracheally challenged aerosol-exposed, P6-immunized mice with live Nontypeable *Haemophilus influenzae* (NTHI) bacteria 8 weeks after vaccination and measured pulmonary bacterial clearance at various time-points post-acute challenge (Fig. 8A). Kinetics of NTHI clearance from lungs was slower in HTP- and EC-exposed P6-vaccinated mice compared with the air-exposed P6-vaccinated controls (Fig. 8B,* p* < 0.05). CC-exposed mice had reduced NTHI clearance compared to HTP-and EC-exposed groups (*p* < 0.01 at 4 h and *p* < 0.0001 at 12 h, respectively) (Fig. 8B), with a CC > HTP > EC > air hierarchy of suppression of bacterial clearance in the lungs. Exposure to HTP and EC exacerbated the accumulation of infection-induced total proteins in the BAL at all time points (*p* < 0.05) and albumin levels at 4 and 12 h following acute challenge compared to air (*p* < 0.01) (Fig. 8C, D). A product hierarchy of CC > HTP = EC > air was observed in the augmentation of the levels of both these markers in the BAL.

## Discussion

Our study provides evidence on how chronic inhalation of aerosols generated from two alternative tobacco products, HTP and EC, impacts the pulmonary inflammatory responses and efficacy of vaccine-induced antibacterial immunity. Importantly, to answer the question of whether those two emerging tobacco products can be considered as potentially harm-reducing alternatives for people who smoke CC, we have compared responses caused by HTP and EC to the effects of CC smoke. Since no reports have directly compared the pulmonary effects HTP vs. EC, our studies were also designed to address this critical gap in knowledge. Generally, the observed induction of detrimental effects associated with chronic exposure to three tobacco products tested in our study followed a CC > HTP > EC hierarchy. However, it is worth noting that in many cases, a proinflammatory pulmonary milieu induced by HTP was comparable to that induced by CC.

As observed in our study, the augmentation in the levels of inflammatory cytokines/chemokines likely mediates HTP-induced pulmonary immune-cell infiltration of leukocytes, neutrophils, and macrophages in a manner equivalent to or greater than EC. While CC overall inflicted more significant damage than both alternative tobacco products, the proinflammatory effects of chronic HTP inhalation were often comparable to that of CC. We are cognizant that the chemical composition of EC and HTP aerosols differs from that of CC smoke, which could play a critical role in the differential accumulation/recruitment of immune cells in the lungs [15–21]. Emissions from HTPs contain numerous chemicals that are also found in CC smoke, and which are classified as highly toxic, e.g., diacetyl, 2,3-pentanedione, hydroxymethylfurfural, and diethylhexyl phthalate [11]. Potentially toxic furans, which are byproducts of the thermal decomposition of sugars, and pyridines, which are produced following the thermal decomposition of nicotine, were detected in the emissions from HTPs and CCs, but are not commonly seen in emissions from ECs [12]. Despite generally lower emission of toxicants from ECs and HTPs compared to CCs, concerns have been raised about respiratory health effects resulting from exposure to toxic carbonyl compounds (incl. formaldehyde, acetaldehyde, and acrolein), which are thermal decomposition of humectants used in HTPs and ECs [13]. Aerosols from ECs often contain high concentrations of common flavoring chemicals (incl. benzaldehyde and cinnamaldehyde), which are known airway irritants and sensitizers and have been reported to cause occupational asthma [14].

The accumulation of neutrophils in the lungs in response to EC and HTP exposure can have significant consequences, as these cells are the source of MPO and NE [26, 27]. MPO is an important inflammatory and oxidative stress marker in several conditions, including lung injury [28, 29]. Augmentation in neutrophilic infiltration and increased NE activity with resulting disruption of lung epithelial barrier integrity is one of the mechanisms through which CC induces pulmonary injury [27, 30, 31]. In this context, the neutrophil and macrophage infiltration induced by emissions from alternative tobacco products could have accounted for the enhanced levels of MPO and NE we detected and likely resulted also in augmented pulmonary oxidative stress.

Previous studies have shown that CC increases susceptibility to respiratory infections by compromising antibacterial immunity [7–10]. Since vaccine-induced immunity is necessary to prevent infections, CC has been demonstrated to reduce the efficacy of prophylactic vaccines [8, 10, 32]. We have previously demonstrated that NTHI P6 protein as a vaccine candidate protects mice from an acute NTHI challenge [8, 10, 33]. Our results clearly show that chronic exposure to aerosols emitted from EC and HTP suppress the production of systemic and mucosal antigen-specific antibodies in a manner akin to CC. Mucosal IgA antibodies, a critical first line of defense against respiratory pathogens, were also significantly diminished following EC and HTP exposures. Suppressed efficacy of P6 vaccination translated into the reduced pulmonary clearance of NTHI bacteria after an acute challenge and led to augmented infection-induced infiltration of neutrophils or macrophages and exacerbated lung damage. These detrimental effects were similar to those seen after CC exposure and similar to previous findings [8, 10, 34]. Following these exposures, a hierarchy of suppression of the efficacy of P6 vaccination and subsequent reduction of bacterial clearance from the lungs of vaccinated mice was found as CC > HTP > EC.

Although the exact mechanisms contributing to EC- and HTP-induced pulmonary inflammation and increased bacterial burden in the lungs have not been established, they could be similar to CC-induced effects. As reported for CC and EC, a potential mechanism could include impaired phagocytic activity [34–36]. Those studies suggested that CC and EC impaired the phagocytic activity of granulocytes and monocytes, contributing to bacterial colonization and increased pathogen burden in the respiratory mucosa, factors that might associate with dysbiosis of the lung microbiome, poor airway health and increased infections in COPD patients [37–41]. While immunosuppressive agents in these products are largely unknown, an analysis of individual constituents in these products and their contribution to inflammatory changes, damage, and immune suppression would be informative. Chung et al. demonstrated that significant immunosuppressive effects could be mediated by nicotine [41]. Importantly, in our study, the exposure protocol for the three products was calibrated so that an equivalent dose of nicotine was delivered to all mice; thus, the differential effects observed are likely unrelated to the differential nicotine intake from tested products. Previous findings [7–10] and our current results support the conclusion that the increased bacterial burden in the lungs of mice exposed to EC or HTP aerosols could be due to impaired innate immune defenses and decreased efficacy of vaccine-induced immunity, including suppressed mucosal antigen-specific IgA production.

An essential strength of our study is the use of precisely adjusted experimental conditions that resulted in animals being exposed to equivalent doses of nicotine from three different tobacco products. This approach eliminated potential confounding effects of differences in physical properties of aerosols emitted from three products that may have affected nicotine delivery to lungs, incl. particulate concentration and aerodynamic particle size. Despite different puffing topographies and different airborne concentrations of nicotine achieved from the three products, our experiments led to similar nicotine exposure across all experimental conditions. This innovative approach allowed us to compare the effects of tested products in realistic conditions, reflecting observations from human studies showing that people who use alternative tobacco products have comparable exposure to nicotine to people who smoke CC [42–45]. Importantly, higher levels of cotinine, a major nicotine metabolite, were observed in female mice compared to male mice in our experiments. This is consistent with the sex-dependent pharmacokinetics of nicotine metabolism in mice, as several studies have shown a faster rate of nicotine elimination from the liver of female than male mice [46–48].

Our study has several limitations. Although we selected popular brands of EC and HTP, it remains to be determined whether our findings can be generalized to other types and brands of alternative tobacco products. Since we performed whole-body exposures, our findings may differ from studies following nose-only exposures. However, our routine sampling did not detect nicotine deposited on animal fur, thereby indicating that inhalation was a primary route of exposure. Since our study directly compared the effects of continued exposure to single products, we did not test the effects of switching between products (e.g., from CC to EC or from CC to HTP). This type of “switching studies” are essential since they are relevant to real-life scenarios when smokers switch to alternative products to reduce the harm associated with smoking CC. However, Husari et al. recently reported that substituting 50% of daily CC exposure with either EC or HTP exposure did not significantly attenuate acute lung injury in a mouse model [49]. Finally, studies are needed to evaluate the effects of concurrent exposures to two products (CC plus EC and CC plus HTP) since epidemiological studies have consistently shown high rates of concurrent use of multiple tobacco products [50–54].

## Conclusions

**Fig. 9:**
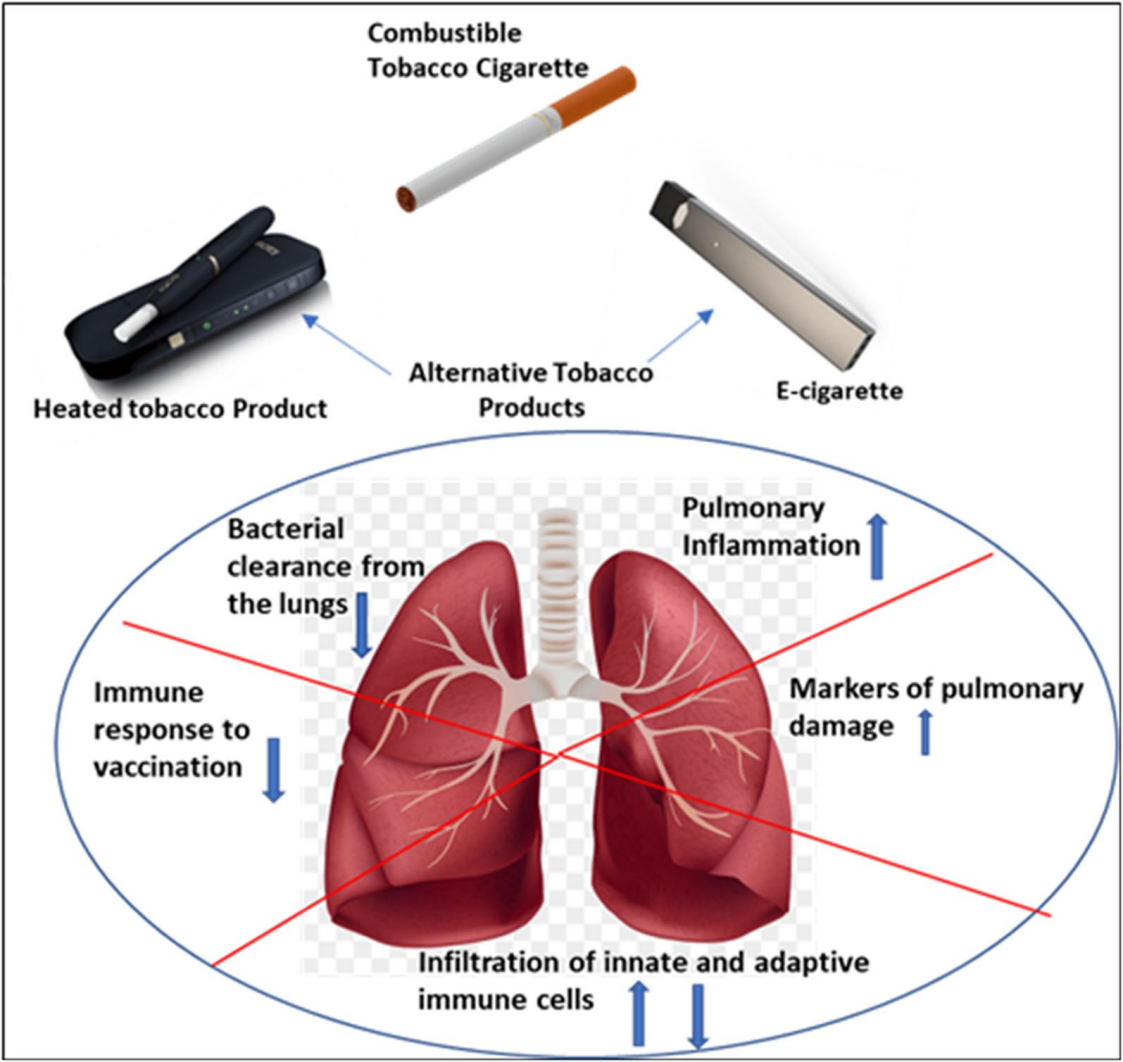
Graphical depiction of alternative tobacco product aerosol-induced detrimental pulmonary effects. The cartoon shows the pulmonary effects induced after chronic exposure to aerosols from alternative tobacco products, HTP and EC, as compared to those induced by cigarette smoke. Upward arrows depict increase, while downward arrows show a decrease

While the alternative tobacco product use has become increasingly popular, the pulmonary health effects resulting from the chronic inhalation of aerosols emitted from these products when compared to smoking combustible tobacco cigarettes are unknown. This study provides evidence on how chronic inhalation exposure to aerosols from two alternative tobacco products impacts pulmonary inflammatory responses, induces oxidative stress, lung endothelial and epithelial damage, and suppresses the efficacy of vaccine-induced antibacterial immunity leading to increased pathogen burden in the lungs of exposed mice in a manner like combustible cigarette smoke (as graphically depicted in Fig. 9). Our study has the potential to open up a broader dialog among clinicians and users, by providing key insights to the adverse respiratory health consequences and immunity suppressive effects resulting from the use of alternative tobacco products. While combustible cigarette smoke overall resulted in more damage than both alternative tobacco products, the proinflammatory effects of chronic HTP inhalation were often comparable to that of CC with the recognition that alternative tobacco product use is not risk-free.

### Supplementary Information

**Additional file 1.** Do alternative tobacco products induce less adverse respiratory risk than cigarettes?

## Data Availability

All data relevant to the study are included in the article or uploaded as online Additional file 1.

## Abbreviations

EC: Electronic cigarettes
HTP: Heated tobacco products
CC: Combustible cigarettes
BAL: Broncho alveolar lavage
MPO: Myeloperoxidase
NE: Neutrophil elastase
NTHI: Nontypeable *Haemophilus influenzae*
