# Supplementary material for: Do alternative tobacco products induce less adverse respiratory risk than cigarettes?
Source: Respir Res. 2023 Oct 31;24:261. doi: 10.1186/s12931-023-02568-2 (PMC10617138; doi:10.1186/s12931-023-02568-2)
Supplement: Supplementary file 1 — Additional file 1. Do alternative tobacco products induce less adverse respiratory risk than cigarettes? [file 12931_2023_2568_MOESM1_ESM.docx]

**Supplementary Materials and methods:**

**Alternative Tobacco Products**

We selected brands of EC and HTP based on their popularity at the time of study initiation. Additionally, the selected brand of HTP (IQOS) is the only product authorized by the US FDA and which was available in the US market. To generate aerosols from EC, we used 2ml EGO CE4 tank (Shenzen Kanger Technology Co. Ltd, China) with a resistance of 2.8±0.6Ω and an output voltage of 4.8V, which resulted in delivering power of 8.2±1.5W. EC tank was filled with commercial 24mg/mL nicotine solution in 50:50 (v/v) mixture of propylene glycol and vegetable glycerin (PG:VG) USA Mix tobacco-flavored purchased from House of Vapor (Kenmore, NY). HTP was an IQOS system (model 2.4) with Marlboro Silver HEETS tobacco inserts purchased from Altria (Richmond, VA). CC were reference tobacco cigarettes 1R6F purchased from the University of Kentucky (Lexington, KY). All inserts for HTP and CC were conditioned at 22.0±1.0°C with a relative humidity of 60.0±2.0% for 48 hours before exposure experiments following CORESTA 21 protocol [**1**].

**Animals**

Eight-week-old C57BL/6NCr mice were purchased from Charles River Laboratory (Wilmington, MA, USA) and housed under pathogen-free conditions at Roswell Park Comprehensive Cancer Center (Buffalo, NY) with a light/dark cycle of 12/12 hour. At the end of 8-week exposure (f**igure 1A**; n=10 for air, n=20 for EC, HTP, and CC/group), mice were euthanized, trachea cannulated, and BAL and lungs were harvested. Animals in vaccination group (n=20 for air, EC, HTP, and CC/group) received prophylactic vaccination i.m. against a respiratory pathogen at weeks 5, 6, and 7 after the start of exposures (f**igure 1B**). Vaccination efficacy was measured by quantifying antigen-specific antibody titers in serum (weeks 5-12) and in BAL at euthanasia. Animal procedures were approved by the Institutional Animal Care and Use Committee and complied with all state, federal, and NIH regulations.

**Animal Exposure Conditions**

Mice were exposed to either air, EC aerosol, HTP aerosol or CC smoke 5h/day, 5 days a week for either 8 weeks (group 1) or 12 weeks (group 2) (f**igure 1**). Animals were placed in exposure cages, each cage holding 10 males and 10 females in total four quadrants (5 same-sex animals/quadrant). Mice were rotated clockwise daily to ensure uniform exposure.

We used an ECAG (CH Technologies; Westwood, NJ) vaping machine to generate aerosols from EC. For exposure to air (control), we used the same system but did not connect any EC product. To generate aerosols from HTP and smoke from CC, we used an automatic JB2090 smoking machine (CH Technologies). Aerosols from each product were generated following the Health Canada Intense puffing regiment (puff of 55 ml volume and 2-sec duration taken every 30 secs [**2**] clustered as described in t**able 2**. Freshly generated aerosols were transferred to animal exposure cages using a tubing system and peristaltic pump. After aerosols from a cluster of puffs were delivered to animal exposure cages, filtered air was consistently supplied to the same cages. Exposure conditions were strictly monitored during each experiment and included measurement of airborne total particulate matter (TPM), PM5.0, airborne nicotine, and nicotine deposited on cage surfaces and animal fur.

We decided to expose animals to an equivalent dose of nicotine delivered from all tested products. Nicotine equivalency was determined by quantifying serum cotinine levels (a major nicotine metabolite) in blood samples collected 30 mins post-exposure. Since aerosols emitted from the three tested products had different physicochemical properties, we performed a series of pilot experiments to calibrate the exposure system and develop a unique puffing protocol for each tested product. Thus, despite differences in the puffing protocols used in our experiments, we achieved equivalent exposure to nicotine from all tested products (**table 2**).

**Assessment of Pulmonary Inflammation**

The pulmonary inflammatory microenvironment was determined by quantifying lung immune-cell infiltration and the levels of cytokines and chemokines in the BAL. Protocol for the isolation of total lung leukocytes for flow cytometry experiments is described as below, the list and source of fluorochrome-conjugated antibodies are provided in **table S1** and the gating strategy in **figure S1**. Cytokine and chemokine levels in the BAL were quantified by performing Luminex multiplex cytokine/chemokine assay as described below.

**Isolation of leukocytes from the lung**

Leukocytes from mouse lungs were isolated as reported previously [**3,4**]. Briefly, lung tissue was minced into small pieces in a 60 mm glass-dish using sterile curved scissors and the resulting tissue slurry was mixed and incubated in 1x PBS solution containing 1 mg/ml Type IA-S collagenase and 50 U/ml DNase I (Sigma-Aldrich) and placed on a rotator for 60 min at 37°C. The resultant single cell suspension was filtered using a 40um cell-strainer to remove debris and undigested tissue and centrifuged at 2,000 rpm for 5 minutes at room temperature. Cell pellet was resuspended in 9 mL complete RMPI-1640 media containing 10% FBS and then underlaid with 5 mL Ficoll-Paque (GE Healthcare) and centrifuged with brake off at 1,700 rpm for 20 minutes at room temperature. Leukocytes at the interface were collected, centrifuged as above, and washed in 1x PBS to remove residual Ficoll-Paque and counted.

**Multiplex** **cytokine/chemokine assay**

Levels of cytokines and chemokines in the BAL were quantified by performing Luminex multiplex cytokine/chemokine assay using MCYTOMAG-70K-27 Mouse Cytokine MAGNETIC Kit (Millipore, Cat# MCYTOMAG-70K-27, Lot# xxxx) following the manufacturer’s instructions and as described previously [**5**]. Data acquisition was performed on FLEXMAP 3D (Luminex Corp. Austin, TX, USA). The five-parameter logistic (5-PL) fitted standard curve for determining the concentrations of various analytes was generated using Arigo Biolaboratories website (<https://www.arigobio.com/elisa-analysis>). During data analysis, limit of detection (LOD) was used for any data points with values <LOD.

**Multicolor flow cytometry**

After euthanasia, lungs were harvested, and lung-tissue digested to isolate leukocytes as stated above and reported previously [**3-5**]. Cells were stained with cell type-specific antibodies to determine the numbers and phenotype of various immune subsets by flow cytometry analysis [**3-5**]. Briefly, 0.5 million cells per sample were stained with specific anti-mouse fluorochrome-tagged Abs in 100 uL volume of FACS staining buffer (1% BSA in PBS) for 30 min at 4°C and subsequently washed in FACS buffer before fixing with Cytofix (BD Biosciences, CA). For intracellular staining, cells were treated with permeabilizing solution (BD Biosciences) and then stained with specific antibodies as described previously [**3-5**]. After washing and subsequent fixing, all the samples were resuspended in 100 uL volume of FACS buffer and stored in dark at 4^o^ C until read. Samples were acquired within 24 hours of staining using LSRII-A flow cytometer and the data was analyzed by FlowJo software. List and source of fluorochrome conjugated antibodies is provided in **Table S1** and the gating strategy is shown in (**figure S1**).

**Estimation of lung damage**

After euthanasia, we measured lung epithelial-cell integrity by quantifying in the BAL the total amount of proteins and the levels of albumin built after systemic to bronchoalveolar space leak as described previously [**5**]. Further, lung endothelial integrity was estimated by measuring the bronchoalveolar to systemic leak of intratracheally-instilled FITC-dextran as described previously [**5**]. Since neutrophil activation leads to increased myeloperoxidase (MPO) activity and neutrophil elastase (NE) levels (refs), we measured these markers in the BAL and lung tissue following various exposures following a procedure as described previously [**5**] and mentioned below. Details of each of these methods are given below.

**Total BAL protein determination**

We measured the total proteins in the BAL by BCA protein assay using a kit from Pierce (Cat#23225) as per manufacturer’s instructions. Various known bovine serum albumin concentrations were used to prepare a standard curve to calculate unknown sample concentrations using a straight-line equation.

**Albumin leak measurement**

The levels of albumin in the BAL samples (a surrogate marker of lung epithelial-cell damage) were measured by ELISA using Bethyl Laboratories (Montgomery, TX, USA) reagents. After following the manufacturer’s assay instructions, plates were developed by 3,3’,5,5’-tetramethylbenzidine (TMB) solution from eBioscience Inc. (San Diego, CA, USA), and the absorbance was read at 450 nm in a Synergy H1 Hybrid plate Reader (BioTek) as reported previously [**3-5**].

**FITC-dextran leak**

To evaluate lung endothelial damage, systemic leak of FITC-dextran from bronchoalveolar space was quantified as described previously [**5**]. To do so, 50 μL aliquot of 200 mg/mL FITC-dextran in 1x PBS was instilled in each mouse *via* intratracheal route (10 μg/mouse). An hour later, mice were anesthetized and bled retro-orbitally and plasma collected by centrifugation. FITC fluorescence in the plasma was determined using Exci485/Emi528 wavelengths in a Synergy H1 Hybrid plate Reader (BioTek). To calculate the FITC-dextran levels in a sample, a standard curve was generated using known concentrations of FITC-dextran in 1x phosphate buffer saline. Unknown sample concentration was determined using the slope and intercept measurements from the standard curve using a straight-line curve equation.

**Myeloperoxidase (MPO) assay**

In this assay, MPO in a sample generates HClO (hypochlorous acid) from H_2_O_2_ and Cl^-^ ion that produces taurine chloramine after reacting with taurine. Taurine chloramine reacts with chromophore TNB and eliminates the color at 412 nm wavelength. Therefore, the absorbance measured at 412 nm in a sample is inversely proportional to the amount of MPO present in that sample. To measure MPO activity in our assays, we used MPO activity assay kit from Abcam (Cat No. #ab105136; Abcam®) and followed the manufacturer’s protocol. Briefly, a sample reaction was started either in presence or absence of MPO substrate in a 96-well ELISA plate and incubated at 25^o^C for 1 hour to generate taurine chloramine following the kit’s instructions. The reaction was stopped by adding a stop mix reagent to the plate and incubating the plate for additional 10 minutes at room temperature. After incubation finished, TNB reagent was added to the plate and incubated for 10 minutes at room temperature. The plate was read at 412 nm wavelength in a Synergy H1 Hybrid plate Reader (BioTek) to measure the absorbance of samples. The data were depicted as the difference in the absorbance in a sample reaction carried either in the presence or absence of substrate reagent and calculated as the change in absorbance at 412 nm (∆OD_412nm_ = (OD_412nm_) sample blank (absence of substrate) minus (OD_412nm_) sample (presence of substrate). OD- optical density.

**Neutrophil elastase (NE) assay**

The levels of neutrophil elastase (NE) in the lung tissue and BAL were measured by using Neutrophil Elastase/ELA2 DuoSet ELISA kit (Cat. No. #DY4517-05; R&D Systems) (Minneapolis, MN, USA) following manufacturer’s kit protocol. Briefly, ELISA plates are coated with anti-NE Ab and kept overnight at room temperature. Next day, after blocking and washing the plates, samples or standards in 1x PBS are added to the plate and incubated for 2 hours at room temperature. After washing 3 times, plates are incubated with detection Ab at room temperature for 2 hours. This is followed by washing the plates 3 times, then adding streptavidin-HRP solution and incubating the plate in dark for 20 minutes at room temperature. At the end of incubation period plates are washed 5 times, substrate solution (mixture of H_2_O_2_ and tetramethylbenzidine) is added to each well and plate incubated in dark for 20 minutes at room temperature. A 2N H_2_SO_4_ stop solution is added to each well and the absorbance of the plate is measured at 450 nm in a Synergy H1 Hybrid plate Reader (BioTek). To correct for optical imperfections in the plate, 540 or 570 nm readings were subtracted from 450 nm readings as per kit recommendations.

**Measurement of oxidative stress in mouse BAL**

Inflammatory responses to environmental insults like CC in the lung rapidly modulate pulmonary antioxidant system to promote lung injury [**6,7**]. Thus, we assessed whether chronic exposure to aerosols from IQOS, EC or CC affected pulmonary antioxidant activity. So immediately after euthanasia, BAL was collected. After protein estimation was done, oxidative stress was quantified by calculating the antioxidant potential in the BAL samples using antioxidant measuring kit form Cayman Laboratories (#Cat. No. 709001; Cayman Labs, USA) following manufacturer’s protocol. This assay measures the ability of antioxidants in a sample to inhibit the oxidation of ABTS^®^ (2,2’-Azino-di-[3-ethylbenzthiazoline sulphonate]) to ABTS^®•+^ by metmyoglobin. The amount of ABTS^®•+^ produced can be monitored by reading the absorbance at 750 nm or 405 nm wavelength. Under these reaction conditions, the antioxidants in the sample cause a suppression of the absorbance at 750 nm or 405 nm to a degree which is proportional to their relative concentration. The capacity of antioxidants in a sample to prevent ABTS^®^ oxidation is compared with that of Trolox, a water-soluble tocopherol analogue, and the data are presented as millimolar Trolox equivalents.

**P6 antigen (Ag) immunization**

In group 2 animals, at wks 5, 6 and 7 after commencement of exposures, all mice were immunized i.m. with a 40 ug purified P6 Ag (an NTHI outer-membrane lipoprotein) in PBS as described previously [**8**], and exposures were continued till wk12. Mice were bled retro-orbitally on a weekly schedule and titers of P6-specific antibodies in serum and end-point BAL were quantified by ELISA as described previously [**3,4**] and described below.

**P6 ELISA**

Titers of P6 Ag-specific antibodies were quantified on ELISA plates coated with 3 µg/mL of purified NTHI P6 protein as described previously (3,4,8). Briefly, serial dilutions of weekly serum and endpoint BAL samples from individual mice were added to BSA-blocked, P6 protein-coated 96-well plates, and bound total anti-P6 Igs were detected with HRP-conjugated goat anti-mouse Ig(H+L) secondary antibody (Southern Biotech, Birmingham, AL, USA). Levels of mucosal antigen-specific IgA in BAL fluid were quantified using 1:400 dilutions of BAL fluid. Plates were gently washed after secondary Ab detection time was over and developed with 3,3’,5,5’-tetramethylbenzidine (TMB; for HRP), and absorbance was read at 450 nm in a Synergy H1 Hybrid plate Reader (BioTek).

**Acute pulmonary NTHI challenge**

Acute pulmonary infections were done using a freshly grown culture of NTHI strain 1479, a clinical isolate from a COPD exacerbation, in all the experiments as described previously (Bhat, et al. 2018, 2020). A single intratracheal instillation of NTHI (1 x 10^6^ live bacteria in 1x PBS) was given to each mouse in a final volume of 50 uL. Animals were euthanized at 0, 4 or 12 h post bacterial challenge to harvest the BAL and lungs for evaluating markers associated with acute inflammation.

**NTHI clearance**

Bacterial clearance was performed as described previously [**3, 4, 8**]. Briefly, mice were given an acute NTHI challenge (10^6^ cfus/mouse) *via* intratracheal route and euthanized at 0, 4 or 12 h post-acute challenge. Lungs were immediately harvested, and under sterile conditions homogenates prepared on ice by gentle homogenization in 1 ml PBS. Serial dilutions of lung homogenates were plated onto chocolate agar plates and incubated at 35°C, 5% CO_2_ for 16 h. Next day, NTHI bacterial colonies were counted, and %clearance rates calculated using the formula %NTHI clearance= {[100 ̶ [((no. of bacterial colonies) x (dilution factor) x (total volume (uL) of lung homogenate)) / (volume (uL) of lung homogenate used for plating)]}%.

**
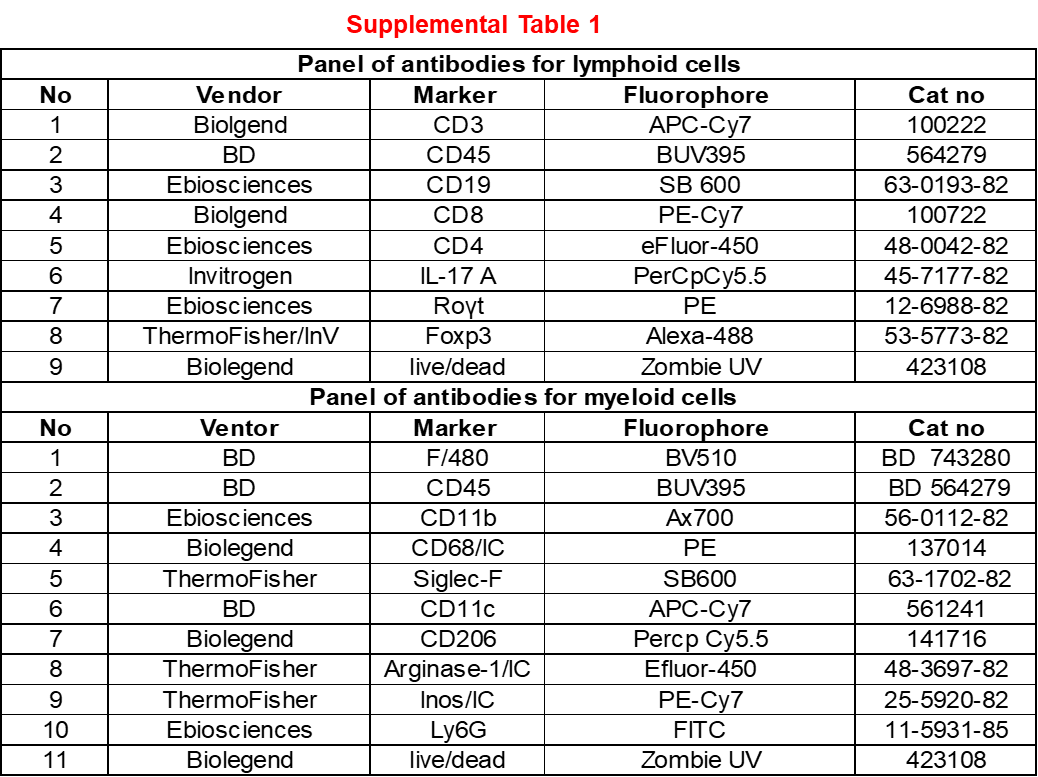
**

**Table S1. Panel of fluorescently labelled antibody markers** specific for various cell phenotypes used to stain samples to perform multicolor flow cytometry and quantify various immune cell subsets in lung tissue of exposed mice.

**References**:

1. ISO 3402:1999. Tobacco and Tobacco Products – Atmosphere for Conditioning and Testing. Geneva, Switzerland: International Organization for Standardization (ISO).
2. ISO/TC 126, Tobacco and tobacco products. ISO and Health Canada intense smoking parameters - Part 2: Examination of factors contributing to variability in the routine measurement of TPM, water and NFDPM smoke yields of cigarettes. ISO/TR 19478-2:2015. [ISO/TR 19478-2:2015(en), ISO and Health Canada intense smoking parameters — Part 2: Examination of factors contributing to variability in the routine measurement of TPM, water and NFDPM smoke yields of cigarettes](https://www.iso.org/obp/ui/#iso:std:iso:tr:19478:-2:ed-1:v1:en).
3. Bhat TA, Kalathil SG, Bogner PN, et al. Secondhand smoke induces inflammation and impairs immunity to respiratory infections. J Immunol. 2018;200(8):2927-2940.
4. Bhat TA, Kalathil SG, Bogner PN, Lehmann PV, Thatcher TH, Sime PJ, Thanavala Y. AT-RvD1 Mitigates Secondhand Smoke-Exacerbated Pulmonary Inflammation and Restores Secondhand Smoke-Suppressed Antibacterial Immunity. J Immunol. 2021 Mar 15;206(6):1348-1360. doi: 10.4049/jimmunol.2001228. Epub 2021 Feb 8. PMID: 33558371; PMCID: PMC7952037.
5. Bhat TA, Kalathil SG, Goniewicz ML, Hutson A, Thanavala Y. Not all vaping is the same: differential pulmonary effects of vaping cannabidiol versus nicotine. Thorax Published Online First: 23 February 2023. doi: 10.1136/thorax-2022-218743
6. Sundar, I.K., Yao, H., Kirkham, P.A., Rahman, I. (2014). Smoking, Oxidative/Carbonyl Stress, and Regulation of Redox Signaling in Lung Inflammation. In: Laher, I. (eds) Systems Biology of Free Radicals and Antioxidants. Springer, Berlin, Heidelberg. <https://doi.org/10.1007/978-3-642-30018-9_65>
7. Foronjy R, D'Armiento J. The Effect of Cigarette Smoke-derived Oxidants on the Inflammatory Response of the Lung. Clin Appl Immunol Rev. 2006 Jan 1;6(1):53-72. doi: 10.1016/j.cair.2006.04.002. PMID: 23997664; PMCID: PMC3755630.
8. Bhat TA, Kalathil SG, Miller A, Thatcher TH, Sime PJ, Thanavala Y. Specialized Proresolving Mediators Overcome Immune Suppression Induced by Exposure to Secondhand Smoke. J Immunol. 2020 Dec 1;205(11):3205-3217. doi: 10.4049/jimmunol.2000711. Epub 2020 Oct 28. PMID: 33115852; PMCID: PMC7686128.


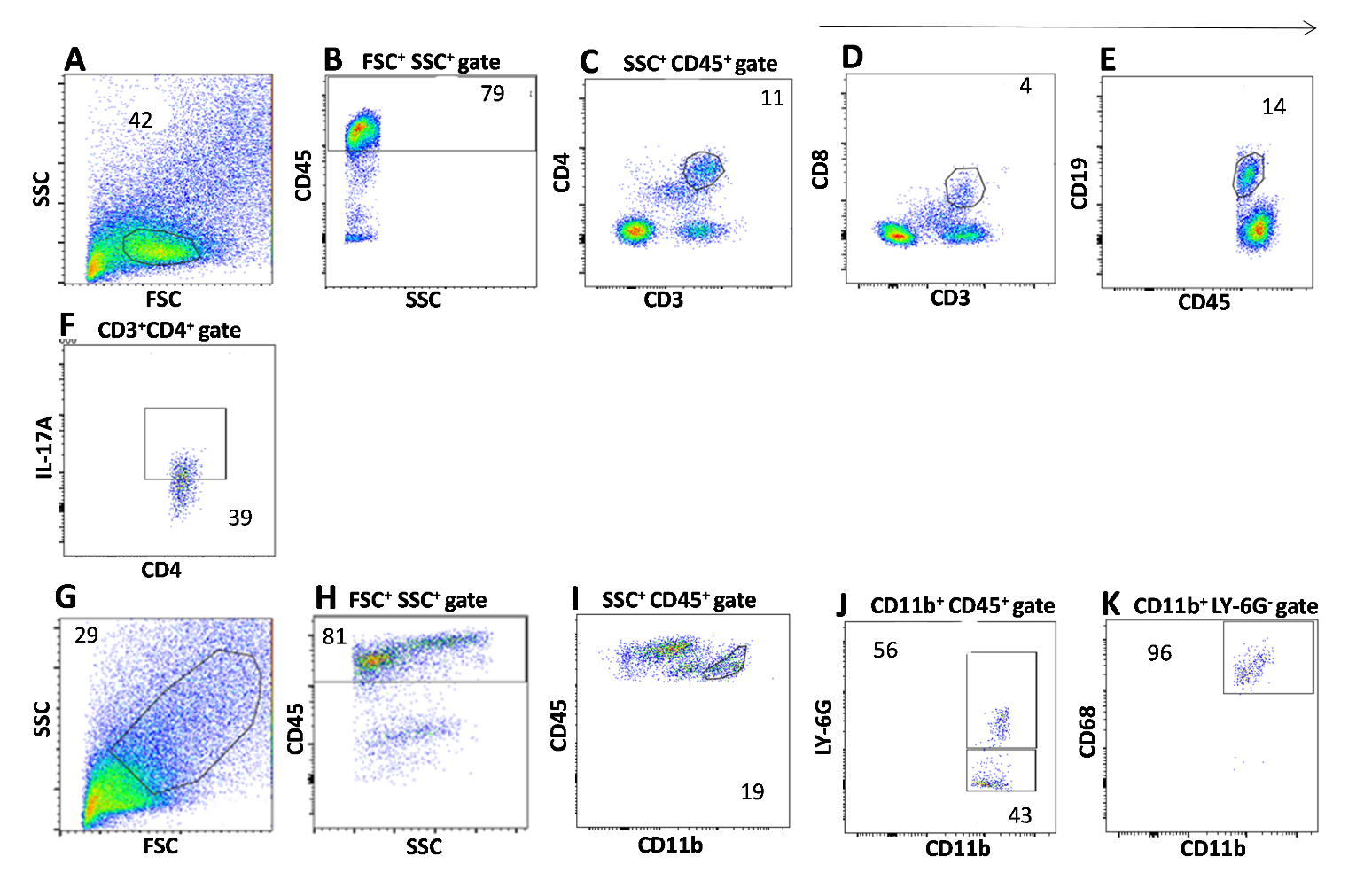
**Figure S1. Representative plots showing the hierarchical gating strategy to analyze lung immune-cell subsets of lymphoid or myeloid origin.** Lung lymphoid cells were gated based on (A) FSC-SSC (B) SSC-CD45^+^ gate and subsequently this population was gated for (C) CD3^+^CD4^+^ T cells (D) CD3^+^CD8^+^ T cells (E) CD45^+^CD19^+^ B cells. CD3^+^CD4^+^ T cells were further gated for (F) IL-17A^+^ CD4^+^ T cells. Furthermore, cells of myeloid origin were subjected to hierarchical gating strategy based on (G) FSC-SSC (H) SSC-CD45^+^ cells (I) CD11b^+^CD45^+^ (J) CD11b^+^Ly6G^+^ neutrophils and CD11b^+^Ly6G^-^ cells further gated for (K) CD11b^+^CD68^+^ macrophages. Numbers indicate the frequencies of respective immune cell subsets gated.


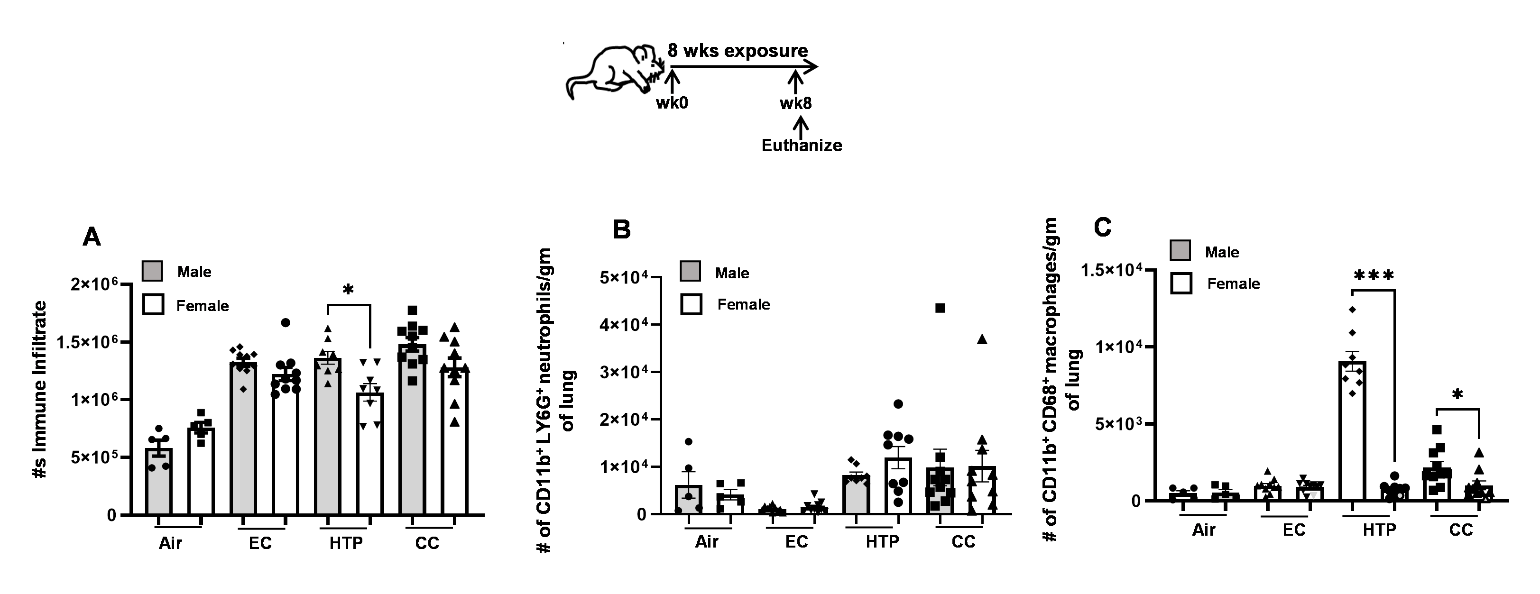
**Figure S2.** **Modulation of innate immune-cells in male *vs*. female mice after alternative tobacco product aerosol-exposure.** Data are presented as bar diagrams with mean ± SE. Non-parametric Kruskal-Wallis test with FDR correction for multiple comparison was performed to see if statistically significant differences exist between two groups using GraphPad Prism V.9 software (GraphPad; La Jolla, California, USA). Difference between two groups is considered significant at p<0.05 and are indicated with symbols *p<0.05; ***p<0.001. n=10/group for each exposure (for air control, n=5 per group).


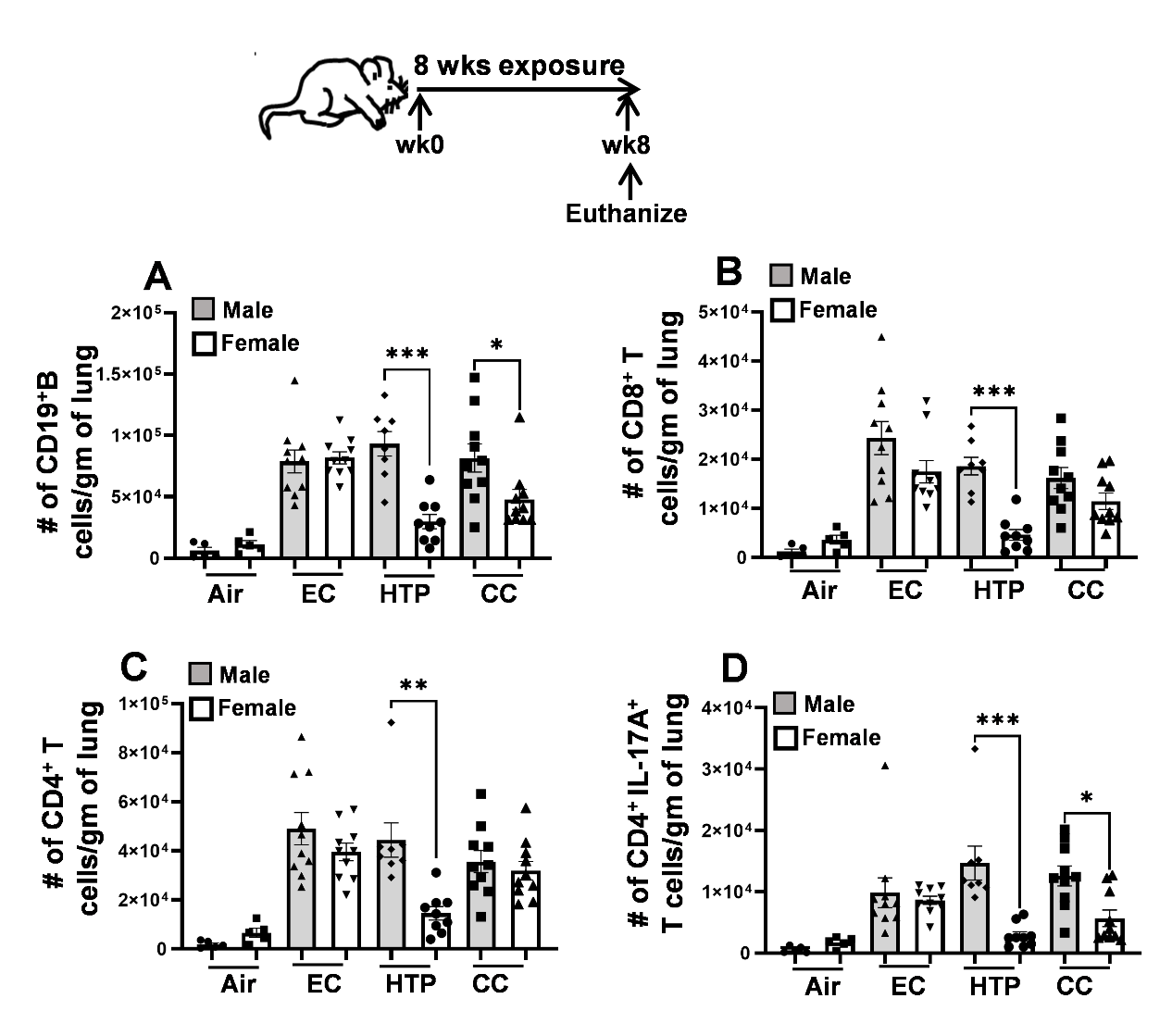
 **Figure S3. Modulation of adaptive and inflammatory immune-cells in male *vs*. female mice following alternative tobacco product aerosol-exposure.** Data are shown as bar diagrams with mean ± SE. Non-parametric Kruskal-Wallis test with FDR correction for multiple comparison was performed to see if statistically significant differences exist between two groups using GraphPad Prism V.9 software (GraphPad; La Jolla, California, USA). Difference between two groups is considered significant at p<0.05 and are indicated with symbols *p<0.05; *p<0.05; **p<0.01; ***p<0.001. n=10/group for each exposure (for air control, n=5 per group).


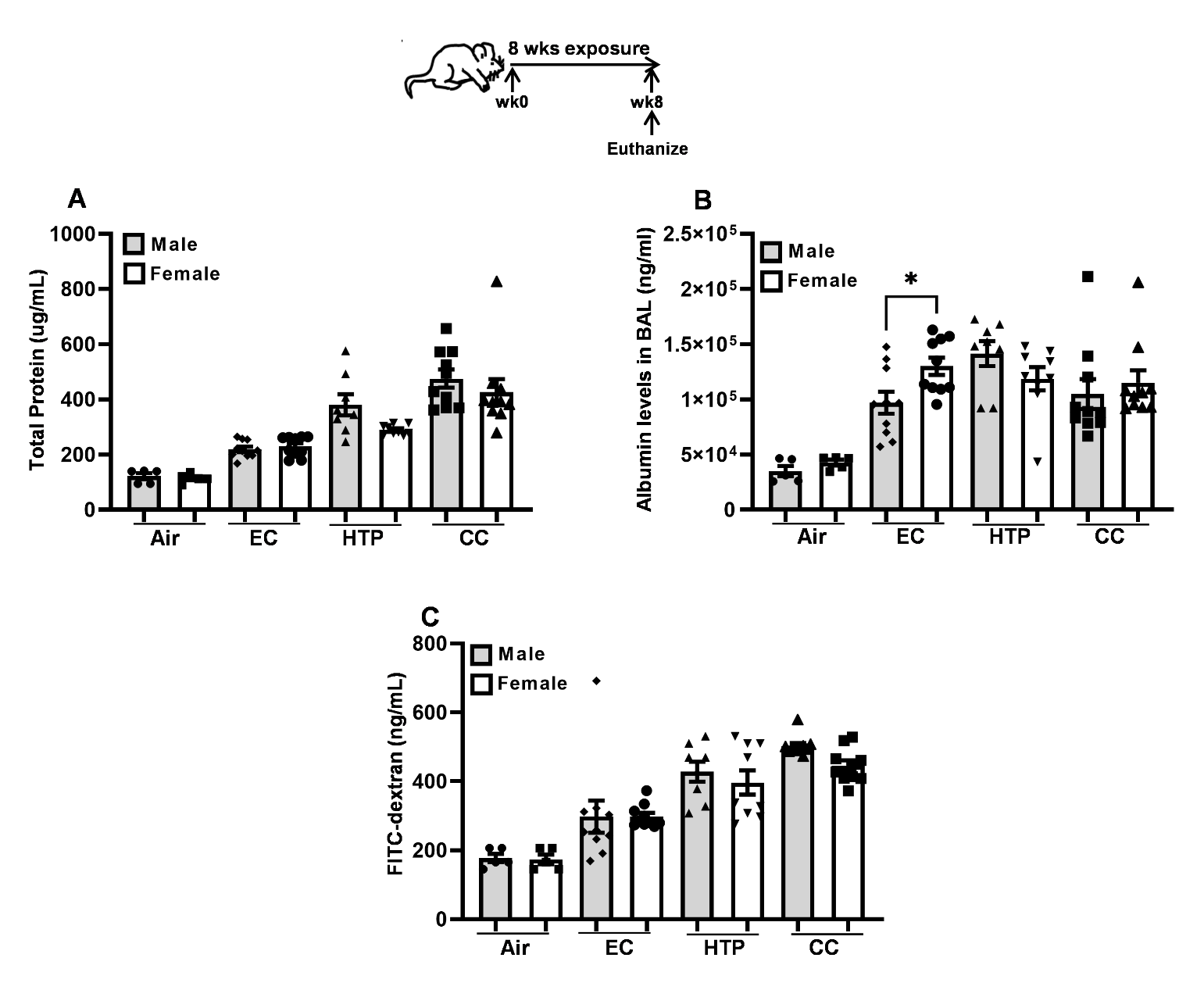
**Figure S4. Markers of lung damage in male *vs*. female mice following to chronic inhalation of aerosols emitted from alternative tobacco products.** Data are given as bar diagrams with mean ± SE. Non-parametric Kruskal-Wallis test with FDR correction for multiple comparison was performed to see if statistically significant differences exist between two groups using GraphPad Prism V.9 software (GraphPad; La Jolla, California, USA). Difference between two groups is considered significant at p<0.05 and are indicated with symbols *p<0.05; *p<0.05; n=10/group for each exposure (for air control, n=5 per group).


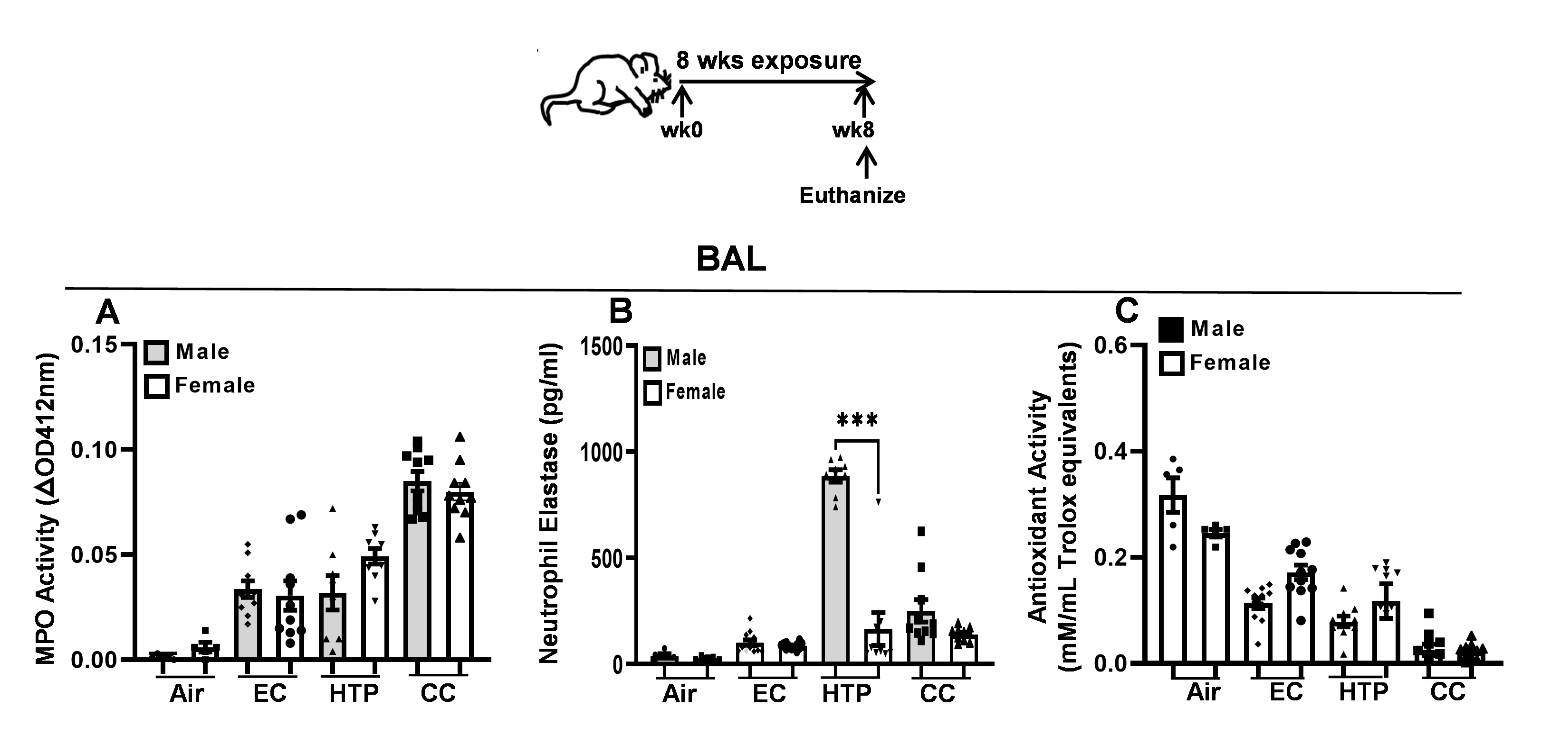


**Figure S5. Modulation of MPO activity, NE levels and antioxidant potential in lungs of** **male *vs*. female mice.** Results are depicted as bar diagrams with mean ± SE. Non-parametric Kruskal-Wallis test with FDR correction for multiple comparison was performed to see if statistically significant differences exist between two groups using GraphPad Prism V.9 software (GraphPad; La Jolla, California, USA). Difference between two groups considered significant at p<0.05; ***p<0.001. n=10/group for each exposure (for air control, n=5 per group).
